# ClC transporter activity modulates histidine catabolism in *Lactobacillus reuteri* by altering intracellular pH and membrane potential

**DOI:** 10.1186/s12934-019-1264-0

**Published:** 2019-12-12

**Authors:** Anne E. Hall, Melinda A. Engevik, Numan Oezguen, Anthony Haag, James Versalovic

**Affiliations:** 1 Department of Molecular Virology and Microbiology, Baylor College of Medicine, Houston, TX 77030 USA; 2 Department of Pathology and Immunology, Baylor College of Medicine, Houston, TX 77030 USA; 3 Department of Pathology, Texas Children’s Hospital, Houston, TX 77030 USA; 4 Present Address: Infectious Disease Laboratories, Akron Children’s Hospital, Akron, OH 44308 USA

**Keywords:** Amino acid, Chloride transport, Decarboxylase, *eriC*, *hdcA*, *hdcP*, Histamine, Lactobacilli, Probiotic, Proton transport

## Abstract

**Background:**

Histamine is a key mediator of the anti-inflammatory activity conferred by the probiotic organism *Lactobacillus reuteri* ATCC PTA 6475 in animal models of colitis and colorectal cancer. In *L. reuteri*, histamine synthesis and secretion requires l-histidine decarboxylase and a l-histidine/histamine exchanger. Chloride channel (ClC)-family proton/chloride antiporters have been proposed to act as electrochemical shunts in conjunction with amino acid decarboxylase systems, correcting ion imbalances generated by decarboxylation through fixed ratio exchange of two chloride ions for one proton. This family is unique among transporters by facilitating ion flux in either direction. Here we examine the histidine decarboxylase system in relation to ClC antiporters in the probiotic organism *Lactobacillus reuteri*.

**Results:**

*In silico* analyses reveal that *L. reuteri* possesses two ClC transporters, EriC and EriC2, as well as a complete histidine decarboxylase gene cluster (HDC) for the synthesis and export of histamine. When the transport activity of either proton/chloride antiporter is disrupted by genetic manipulation, bacterial histamine output is reduced. Using fluorescent reporter assays, we further show that ClC transporters affect histamine output by altering intracellular pH and membrane potential. ClC transport also alters the expression and activity of two key HDC genes: the histidine decarboxylase (*hdcA*) and the histidine/histamine exchanger (*hdcP*).

**Conclusions:**

Histamine production is a potentially beneficial feature for intestinal microbes by promoting long-term colonization and suppression of inflammation and host immune responses. ClC transporters may serve as tunable modulators for histamine production by *L. reuteri* and other gut microbes.

## Background

Bacterial enzymes enable the production of microbial metabolites by the microbiome and confer important effects on microbiome:host dynamics [1]. However, bacterial enzyme activity is often affected by pH, and many metabolite transporters rely on voltage- or ion transport-dependent gating mechanisms [2, 3]. While some bacteria maintain their intracellular pH and the ion gradients across their membranes within narrow boundaries, the lactic acid bacteria (LAB) can tolerate large shifts in these values in response to their extracellular environment [4]. It may be difficult to ensure the optimal intracellular conditions for production of either natural or exogenous products in a heterogeneous environment such as the mammalian gastrointestinal tract [5, 6].

The model probiotic organism, *Lactobacillus reuteri* is a gram-positive LAB that can be found as a commensal organism among many hosts, including birds, pigs, rodents and humans [7]. Members of this species can have varied probiotic effects determined by their strain-level genetic diversity [8–11]. Strains from multiple human-associated clades have been used successfully as probiotics [8–11]. In particular, the human breast milk-derived strain *L. reuteri* ATCC PTA 6475 (also known as strain MM4-1A) has been shown to reduce inflammation in murine models of colitis and inflammation-associated colorectal cancer [12–15]. This strain has also been shown to reduce production of proinflammatory cytokines by primary macrophages isolated from pediatric Crohn’s Disease patients [16] and to diminish antibiotic-associated side effects in *Helicobacter pylori* infected patients [11]. In vitro studies using a human monocytoid cell line demonstrate that the anti-inflammatory effects of *L. reuteri* 6475 are closely linked with the bacterium’s ability to produce histamine [17].

The mechanism of histamine production by lactic acid bacteria has been well described [18]. In our strain of interest, histamine synthesis genes are organized in a cluster consisting of (in order) an l-histidine/histamine exchanger (encoded by *hdcP*), a pyruvate-dependent histidine decarboxylase (*hdcA*), a putative maturation enzyme for HdcA (*hdcB*), and a histidyl-tRNA synthetase (*hisRS2*) [7, 19]. *hdcA* and *hdcB* are co-transcribed as a single RNA, while the other genes in the cluster are transcribed independently [19]. A three-step process occurs for every molecule of histamine produced by *L. reuteri*. First, l-histidine is brought into the cell by HdcP. Next, HdcA cleaves the carboxyl group from the amino acid in a reaction that consumes an intracellular proton and produces histamine and carbon dioxide. Finally, histamine (which now has increased its charge by + 1 over l-histidine) is exported by HdcP via coupled antiport with a new l-histidine molecule. In this way, protons are effectively pumped out of the cell, which may increase intracellular pH and inside-negative membrane potential. As such, amino acid decarboxylase systems are often regarded as part of the bacterial acid resistance response [20, 21]. However, the enzymatic activity of the histidine decarboxylase of lactic acid bacteria is maximal at pH near 4.0, and this enzyme has reduced activity at neutral and alkaline pH. Moreover, the activity of the HdcP histidine/histamine exchanger can be increased by an internally positive membrane potential and inhibited by internally negative membrane potential [22–24]. It is unclear how lactic acid bacteria balance changes in electrochemical gradients required for sustained histamine production.

A random mutagenesis screen located a potential HDC regulator in *L. reuteri* 6475. Knockout of a ClC proton/chloride antiporter EriC2 (previously annotated as EriC) significantly reduced histamine production and altered expression of the HDC gene cluster [25]. However, the exact role for EriC2 in histamine production has not been determined. ClC transporters are highly conserved, and are found in all kingdoms of life from bacteria to humans [26]. Kinetic studies of ClcA from *E. coli* and other homologous transporters suggest that this family of proteins exhibit secondary active transport, exchanging one proton for two chloride ions (1 H^+^/2 Cl^−^) in a fixed ratio with the stronger gradient of one ion driving transport of the other [27–29]. Early investigations of ClcA in *E. coli* suggested that ClC transporters can act as key regulators of amino acid decarboxylase systems, by relieving highly negative membrane potential associated with amino acid decarboxylation through chloride (Cl^−^) export [30]. Other groups have suggested that ClC transporters might act in the opposite way, balancing internally positive potential after acid stress via chloride import, driven by the transmembrane pH gradient [31].

In this work, we sought to characterize the role of ClC-family ion transport in histamine production by *L. reuteri* 6475. We first show that altering extracellular ionic environment alters synthesis of histamine by the native bacteria. Through genetic manipulation, we also demonstrate that ion transport through ClC-family proteins is critical for balancing electrochemical gradients during histamine production, and for enabling amino acid decarboxylation systems to function optimally in the intestinal microbiome. These findings point to the potential for ClC-family H^+^/Cl^−^ antiporters to serve as tunable modulators for basic physiological properties of natural (or engineered) probiotic strains.

## Results

### Extracellular chloride concentration and pH regulate histamine output via proton/chloride antiporters

Previous work indicates that the histidine decarboxylases of lactic acid bacteria, including *Lactobacillus* species, function best at acidic pH [18, 24, 32]. Evidence also suggests that increasing extracellular chloride concentration can inhibit histamine output [33]. Since ClC-family transporters can modulate both intracellular pH and chloride concentration, we examined the effects of the extracellular environment on histamine production by *L. reuteri.* Wild type bacteria were incubated in buffer containing l-histidine at a variable pH (4.5, 5.5, 6.5, or 7.5) and stable chloride concentration [95 mM total Cl^–^], or variable chloride (15, 20, 35, or 95 mM [total Cl^−^]) and stable pH (5.0). pH 5.0 has been shown to be an effective pH for *in vitro* histamine production assays [23, 24]. Histamine in the cell-free supernatants was quantified by liquid chromatography–mass spectrometry (LC–MS). Raw histamine values were normalized to the optical density (OD_600_) of the input culture (N = 4 per group).

**Fig. 1:**
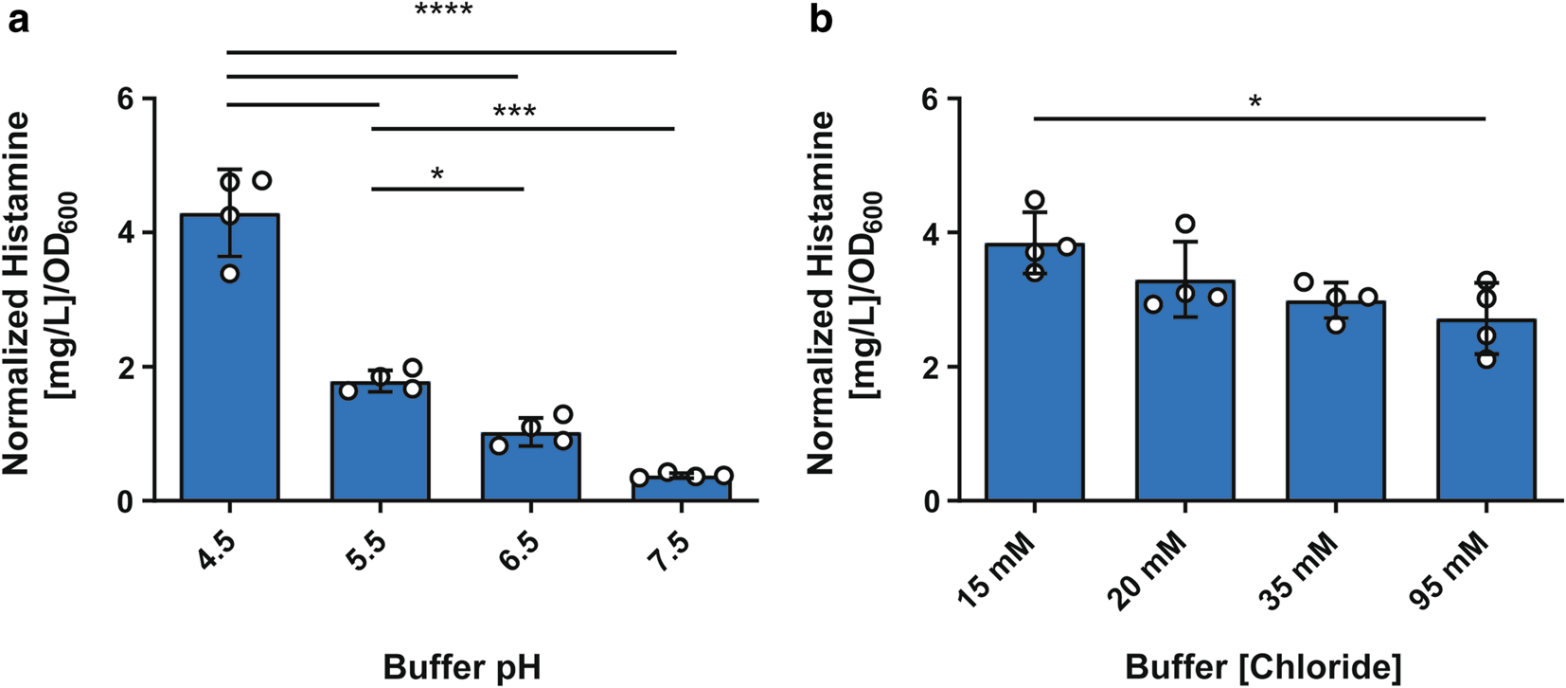
Histamine produced by *wild type* (WT) *L. reuteri* given variable extracellular **a** pH or **b** [chloride]. Cells were cultured in rich media (MRS) for 24 h, prior to washing and resuspension in potassium citrate buffers at **a** pH 4.5, 5.5, 6.5, or 7.5 with 80 mM sodium chloride and 15 mM l-histidine hydrochloride (95 mM total Cl^−^) or **b** pH 5.0 with 0, 5, 20 or 80 mM sodium chloride and 15 mM l-histidine hydrochloride (15, 20, 35, or 95 mM total Cl^−^) for 3.5 h. Histamine in cell-free supernatants was measured by LC–MS. Raw histamine concentrations were normalized to the OD_600_ value in MRS obtained for each sample prior to resuspension. N = 4 biological replicates. Error bars represent ± SEM. One way ANOVA with Tukey’s multiple comparison test. *P < 0.05, ***P < 0.001, ****P < 0.0001

In the variable pH assay, histamine output ranged from 0.376 ± 0.019 mg/L (mean ± SEM) when bacterial cells were incubated at pH = 7.5, to a maximum of 4.293 ± 0.325 mg/L when cells were incubated at pH = 4.5 buffer (Fig. 1a, one-way ANOVA with Tukey’s multiple comparison test, P < 0.05–0.0001). Additionally, histamine output was found to vary inversely with chloride concentration. At the minimum tested value of 15 mM [total Cl^−^], *L. reuteri* produced histamine at a concentration of 3.846 ± 0.230 mg/L, and histamine output was suppressed (yield: 2.718 ± 0.264 mg/L) by increasing the total chloride concentration to 95 mM (Fig. 1b, one-way ANOVA, with Tukey’s multiple comparison test, P < 0.05).

### *L. reuteri* 6475 has two ClcA type proton/ chloride antiporters with highly conserved gating regions

**Fig. 2:**
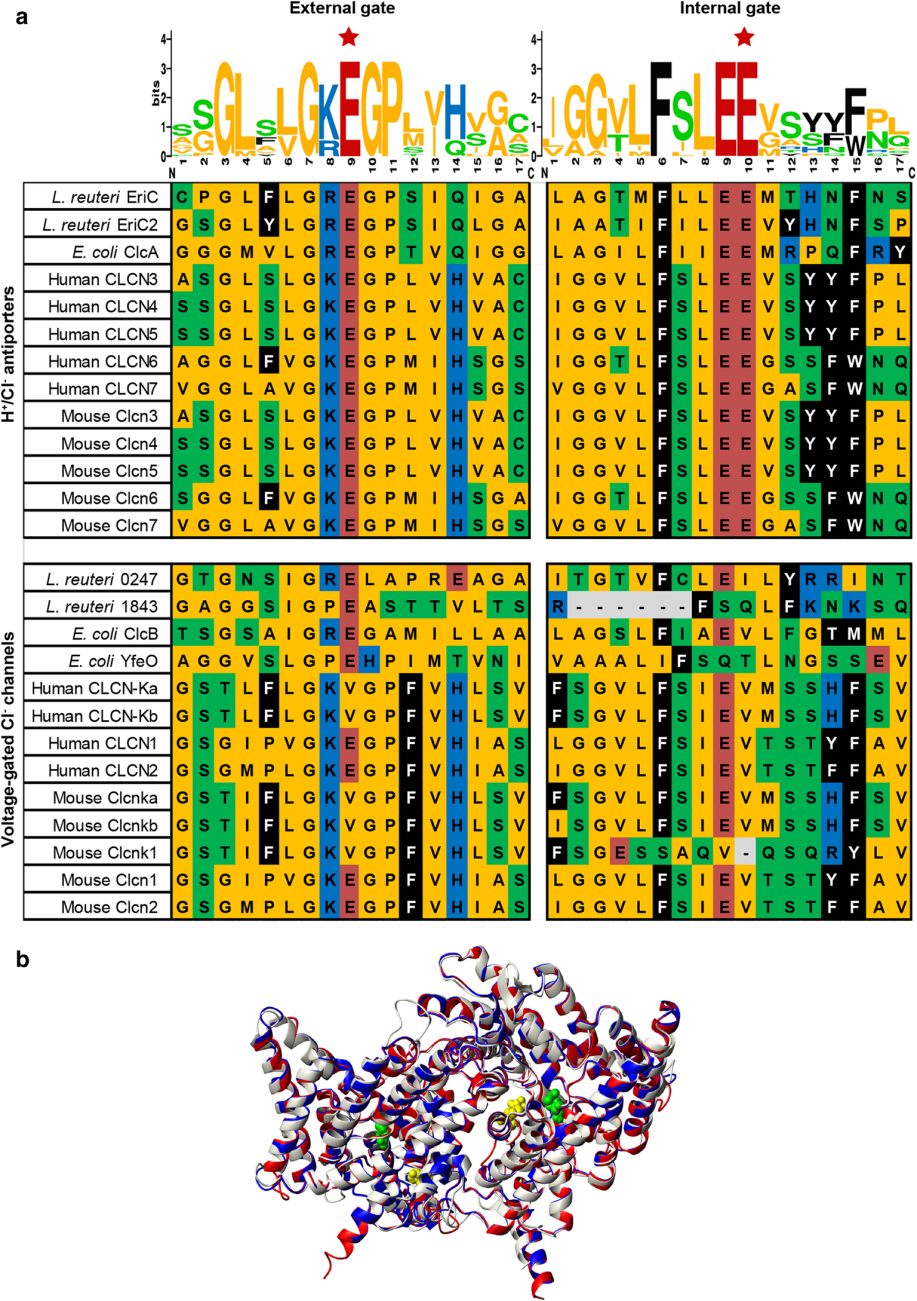
Interkingdom alignment and *in silico* modeling of ClC family antiporters. **a** Amino acid alignments for external (left) and internal (right) gating domains in genes annotated with COG0038 (H^+^/Cl^−^ antiporter ClcA) in *L. reuteri*, *E. coli*, mouse, and human (see Additional file 3: Table S3 for gene/genome identifiers). Antiporters and channels are grouped separately and sequence logos (top) denote the conservation of each residue in the antiporter class. Gating glutamate residues are marked (★). Residues are colored by side chain chemistry (aromatic—black, acidic—red, basic—blue, nonpolar—yellow, polar—green). **b** The ribbon model of ClcA is shown in red, overlapped with models for EriC (white) and EriC2 (blue). Gating glutamate residues are displayed as space-filling atomic surfaces. The external gate is displayed in green (ClcA E148, EriC E144, EriC2 E139), and the internal gate is highlighted in yellow (ClcA E203, EriC E199, EriC2 E193). Models were generated with SwissModel, and visualized with MOLMOL

ClC transporters exhibit a high degree of conservation across kingdoms, particularly at the internal and external gating regions required for coupled proton/chloride antiport. By searching the functional annotations of the *L. reuteri* 6475 genome for the ClC proton/chloride antiporter (COG0038) we identified four potential genes of interest in *L. reuteri*, and compared them to their homologs from *E. coli,* human, and mouse. The alignments in Fig. 2a demonstrate that only two of these genes (EriC and EriC2) are true antiporters as evidenced by the presence of the gating glutamates in both the external and internal domains. Natively, these proteins function as homodimers, with each pore able to carry out independent proton/chloride exchange. Using an *E. coli* template (ClcA, PDB: 1kpk), we generated in silico 3D structural models for the amino acid sequences of EriC and EriC2. For both proteins the overall sequence identity to the template for the modeled regions was 29% (data not shown). However, the sequence identity for the ± 10 amino acids around the internal and external glutamates was 52% and 43%, indicating greater reliability of the models for the two regions of interest (Fig. 2b).

Previous work using *E. coli*’s ClcA demonstrated that replacement of the external glutamate for an alanine is predicted to lock the gate in an “Open” position, allowing gradient-dependent motion of chloride ions, but prohibiting proton transport [28]. Conversely, alanine substitution at the internal gate prevents movement of both protons and chloride ions, such that the gate is effectively “Closed” [34, 35]. We introduced these targeted substitutions into *L. reuteri*’s EriC and EriC2 proteins individually, and in combination, to test how transport activity might affect the production and export of histamine. We compared these targeted mutants to ones with early “Stop” codons, generating targeted inactivation of functional protein. This suite of nine ion transport mutants (EriC Open, EriC Closed, EriC Stop, EriC2 Open, EriC2 Closed, EriC2 Stop, Double Open, Double Closed, and Double Stop) was used throughout the following studies, and these results were compared to data obtained with wild type *L. reuteri*, and *L. reuteri* strains lacking a functional histidine decarboxylase (HdcA Stop) or histidine/ histamine exchanger (HdcP Stop).

None of the introduced mutations affected the overall growth pattern of the strains, as evidenced by the optical density of each culture in rich media (de Man-Rogosa-Sharpe medium-MRS; Additional file 1: Figure S1A; two-way repeated measures ANOVA, with Dunnett’s multiple comparison test relative to Wild Type; minimum P < 0.001; N = 3 per strain). Some mutations contributed to slightly increased survival in late stationary phase (Additional file 1: Figure S1B; two-way repeated measures ANOVA, with Dunnett’s multiple comparison test relative to WT; minimum P < 0.01).

### Disruption of ion transport via EriC or EriC2 results in reduced histamine production

**Fig. 3:**
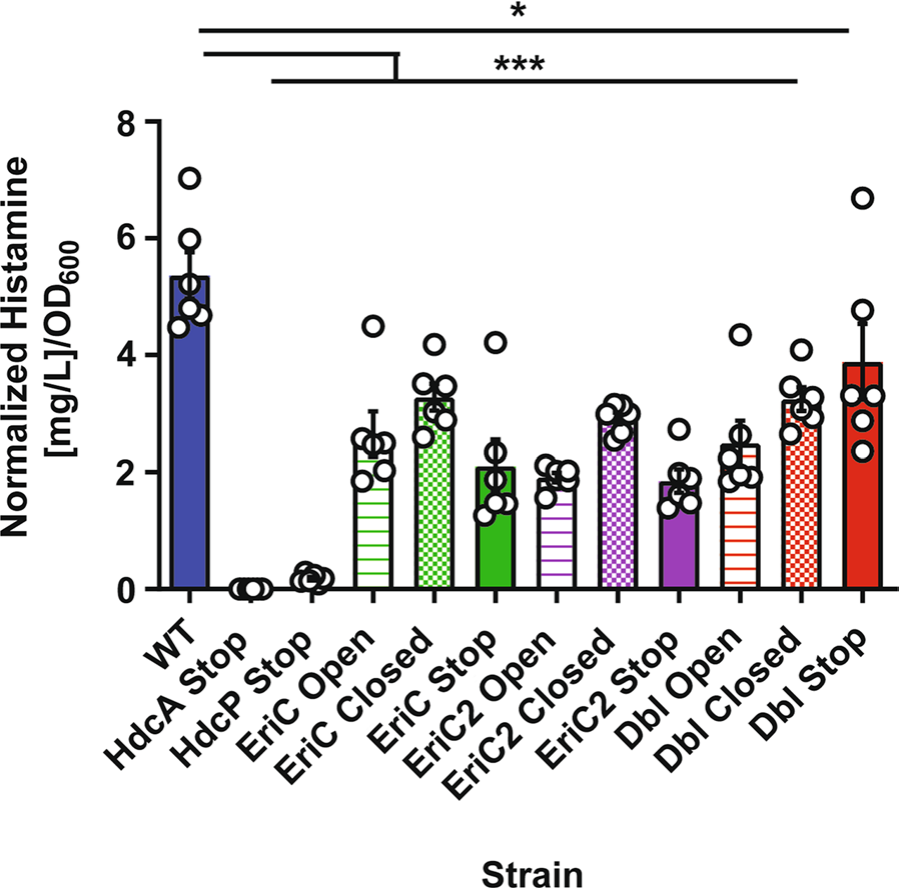
LC–MS quantitation of histamine produced by *wild type* (WT) and mutant *L. reuteri* strains. Cells were cultured in rich media (MRS) for 24 h, prior to washing and resuspension in buffer (pH 5.0) with l-histidine for 3.5 h. Raw histamine concentrations were normalized to the OD_600_ value in MRS obtained for each sample prior to resuspension. N = 6 biological replicates. Error bars represent ± SEM. One way ANOVA with Dunnett’s multiple comparison test relative to WT. *P < 0.05, ***P < 0.001

We next assessed the ability of our *wild type* and mutant strains to produce and secrete histamine in buffer (pH 5.0, 80 mM NaCl, 15 mM l-histidine). Histamine in the supernatant was quantified by LC–MS, and normalized by absorbance spectrophotometry (OD_600_) of the input culture (Fig. 3). One-way ANOVA with Dunnett’s multiple comparison test relative to the *wild type* strain was used to assess differences among the mutant strains (N = 6 per strain). The wild type strain produced the most histamine, as expected (5.364 ± 0.398 mg/L). As predicted, the strain lacking an intact *hdcA* gene did not yield detectable histamine, while the strain lacking the intact *hdcP* transporter gene secreted histamine at a concentration several logs lower than the wild type strain (0.180 ± 0.030 mg/L, P < 0.001). All EriC and EriC2 mutant strains produced significantly less histamine than the wild type strain (Range: 1.850 ± 0.201 to 3.884 ± 0.650 mg/L, P < 0.05 to < 0.001). Among single mutations of EriC or EriC2, the effect is most pronounced when the gene is fully inactivated, followed by locking the antiporter in the open state. Among the single mutants, we might have expected “Closed” mutants and “Stop” mutants, to react similarly, as they are both functionally inactive. However, “Closed” mutants seems to produce slightly more histamine. This may be due to the fact that the “Closed” channels may be “leaky” to ion movement compared to “Stop” mutants, where no protein is synthesized [28, 35]. The effect is least pronounced when the antiporter is locked in the closed state. A single amino acid substitution in the gating mechanism appears to be sufficient to reduce histamine output. This finding indicates that these transporters likely exert their effects on the histamine synthesis system as functional modulators of the external environment, rather than by any other mechanism, such as binding directly to DNA or other proteins. Paradoxically, we do not see a synergistic effect in strains with dual inactivation of the EriC and EriC2 genes. This result might might indicate an additional compensatory mechanism that is active in the absence of both functional transporters.

### Ion transport disruption alters expression of the HDC and ClcA-family transporters

**Fig. 4:**
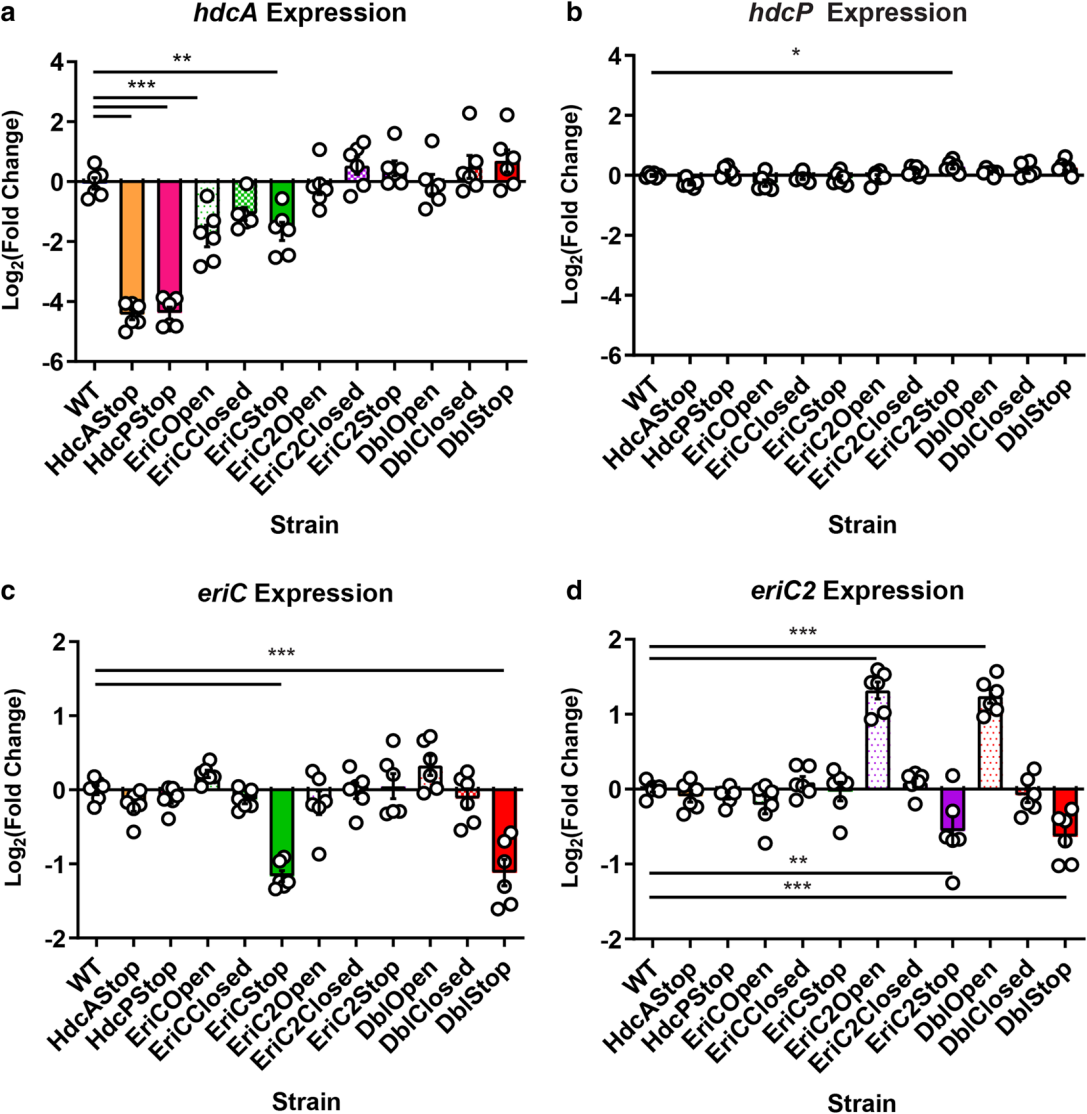
Expression of histidine decarboxylase (*hdcA*), histidine/histamine antiporter (*hdcP*), and proton/chloride antiporters (*eriC/eriC2*) in *L. reuteri*. cDNA was synthesized from RNA extracted from 24 h MRS cultures. Expression values are reported as fold changes in cycle threshold (Ct) using the 2^−ΔΔCt^ method [36]. In each assay, *rpoB* (RNA polymerase, β subunit) was used as the reference gene and the Ct difference between the gene of interest and the reference gene in the *wild type* strain was used as the reference condition for all comparisons. Data displayed as the mean Log_2_ of the fold change value (−ΔΔCt), ± SEM. N = 6 biological replicates. One-way ANOVA with Dunnett’s multiple comparison test compared to WT. *P < 0.05, **P < 0.01, ***P < 0.001

Next we profiled the expression of the prominent HDC genes, *hdcA* and *hdcP*, as well as the two ClC-family transporters themselves, *eriC* and *eriC2* in wild type and mutant *L. reuteri* by qPCR (Fig. 4). Fold changes in cycle threshold were determined between genes of interest and the housekeeping gene *rpoB* (RNA polymerase, β subunit) using the 2^−ΔΔCt^ method [36]. One-way ANOVA with Dunnett’s multiple comparison test was used to detect deviation from the wild type (N = 6 per strain). Expression of the histidine/histamine exchanger, *hdcP* is relatively unchanged in all strains, with the exception of a statistically significant 2^0.29^-fold upregulation in the HdcP Stop strain (P < 0.05). Expression of the *hdcA* histidine decarboxylase is significantly downregulated in the HdcA and HdcP stop strains (2^−4.44^ and 2^−4.38^-fold respectively, P < 0.001), as well as the EriC Open and EriC stop strains (2^−1.81^- and 2^−1.66^-fold respectively, P < 0.001 and P < 0.01). Expression of *eriC* is significantly downregulated in strains where it has been inactivated, EriC Stop and Dbl Stop (2^−1.17^- and 2^−1.12^-fold, P < 0.001). Interestingly, *eriC2* exhibits a unique pattern. Expression of this gene is upregulated in strains carrying the EriC2 Open mutation (EriC2 Open and Dbl Open, 2^1.32^- and 2^1.24^-fold, P < 0.001), and downregulated where EriC2 has been inactivated (EriC2 Stop and Dbl Stop, 2^−0.56^- fold and 2^−0.63^-fold, P < 0.01 and P < 0.001). Taken together these data suggest that the decreased histamine output by transporter mutant strains is not likely due to the downregulation of HDC expression. In addition, these data suggest that EriC and EriC2 are differentially regulated in *L. reuteri*, despite their high degree of homology.

### Intracellular pH is altered by mutations in EriC or EriC2

**Fig. 5:**
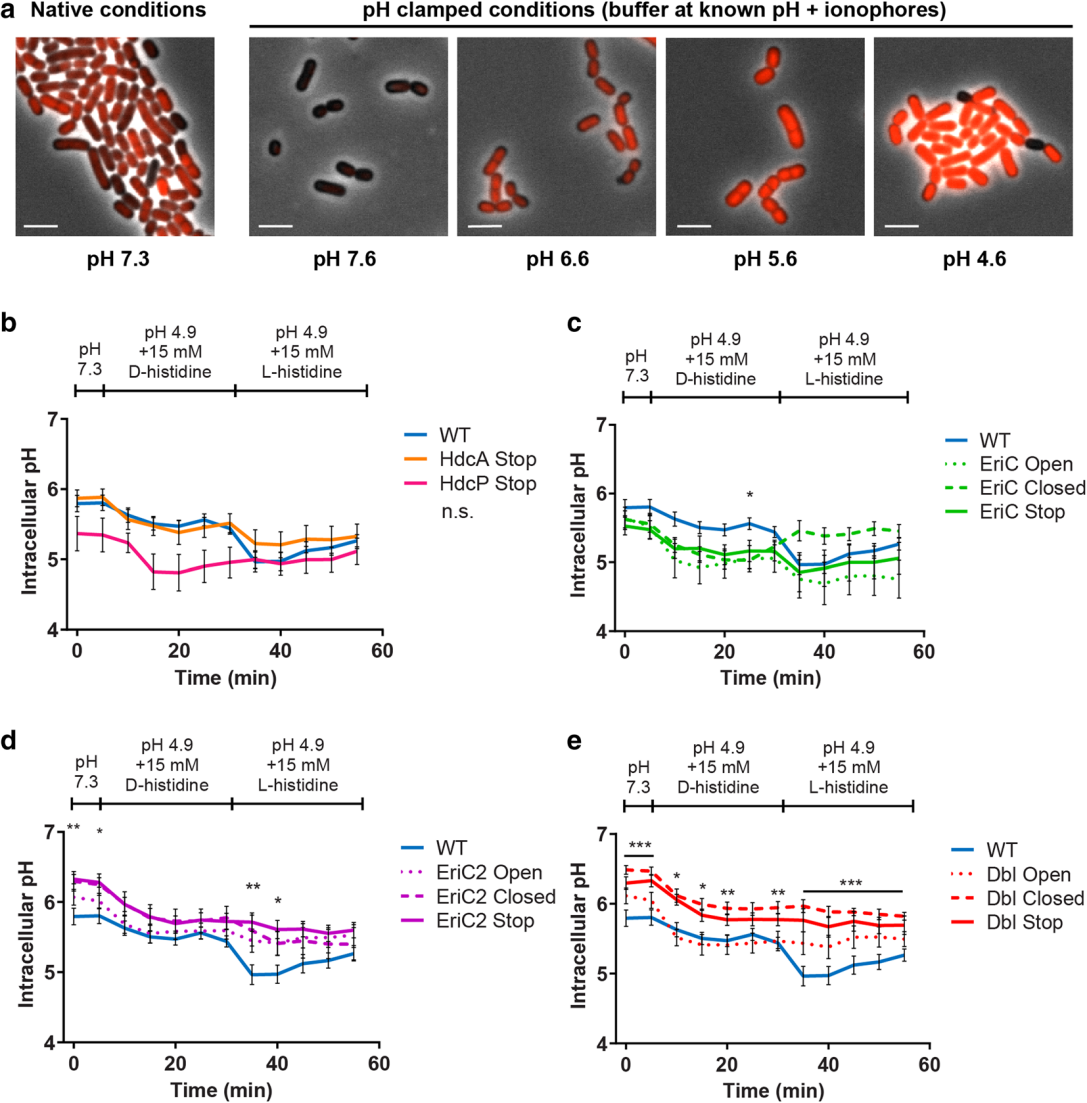
Intracellular pH measurement in WT and mutant *L. reuteri* strains during histamine production. Cells were grown for 24 h in MRS prior to washing and loading with pHrodo Red pH sensitive dye. **a** Representative images from an intracellular pH standard curve using pHrodo Red. Native stained cells exhibit slight intercellular variation (left panel), but intracellular pH can be equilibrated to the extracellular pH using ionophores (right panels). Image scale bar indicates 2 µm. Cells were grown for 24 h in MRS prior to washing and loading with pHrodo Red pH sensitive dye. For quantitative measures, fluorescence of cells was monitored in a plate reader at pH 7.3, pH 4.9 + d-histidine, and pH 4.9 + l-histidine. Graphs above depict fluorescence traces of the *wild type* strain compared to **a** HdcA and HdcP knockout strains, **b** EriC mutant strains, **c** EriC2 mutant strains, and **d** double EriC/EriC2 mutant strains. Error bars represent ± SEM. Indicated P-values are from a two-way repeated measures ANOVA with Dunnett’s multiple comparison test (*P < 0.05, **P < 0.01, ***P < 0.001, *n.s.* not significant)

We predict that ClC-family proton/chloride antiporters affect histamine production through modulation of the internal ionic environment. As such, we set out to measure the intracellular pH (pH_i_) and membrane potential (Δψ) in our suite of *L. reuteri* mutant strains. Changes in pH_i_ were monitored over time using the pH-sensitive fluorophore pHrodo Red (Fig. 5a, Additional file 2: Figure S2) as the extracellular medium was acidified in the presence of d-histidine (which cannot be metabolized to histamine), and then as d-histidine was replaced with l-histidine (Fig. 5b–e). A two-way repeated measures ANOVA with Dunnett’s multiple comparison test was used to determine if pH_i_ was different in mutant strains compared to the wild type strain during this treatment (N = 12 per strain).

During the assay, the pH_i_ of the *wild type* strain varies from a maximum of 5.80 ± 0.11 to a minimum of 4.96 ± 0.14. This strain acidifies only mildly at the exposure to a drop in extracellular pH when d-histidine is present (Fig. 5b). When l-histidine is made available, the pH_i_ is further diminished. We attribute the initial drop in intracellular pH upon histamine synthesis to the production and dissolution of carbon dioxide in this aqueous system [37]. As histamine is exported and CO_2_ outgasses, intracellular pH climbs. Over the assay period, pH_i_ is not significantly different from wild type values for the HdcA or HdcP Stop mutants. However, we did observe a interesting trends with the HdcP loss-of-function mutant, in which pH_i_ acidifies to its minimum (4.81 ± 0.26) immediately upon acid exposure, but does show some correction over time. The HdcA mutant follows the wild type trace more closely in the absence of l-histidine but does not appear to acidify to the same degree as the wild type (minimum pH_i_ 5.21 ± 0.13). Taken together, these data illustrate the importance of histidine uptake and histamine synthesis for maintaining pH_i_ during extracellular acid exposure.

The overall trend in our data suggests that mutations in EriC decrease intracellular pH (minimum pH_i_: EriC Open, 4.69 ± 0.31; maximum pH_i_: EriC Open 5.64 ± 0.16; Fig. 5c), while mutations in EriC2 increase intracellular pH (minimum pH_i_: EriC2 Closed, 5.40 ± 0.24; maximum pH_i_: EriC2 Stop 6.33 ± 0.07; Fig. 5d) compared to the *wild type*. The pH_i_ values of the double mutant strains tracks with the comparable EriC2 strains (minimum pH_i_: Dbl Open, 5.38 ± 0.17; maximum pH_i_: Dbl Closed 6.49 ± 0.06; Fig. 5e). Interestingly, pH_i_ begins to increase just before the change to l-histidine in the EriC Closed mutant, indicating that this strain may be activating a compensatory acid control mechanism independent of HDC machinery. These data demonstrate that ClC-family transporters play a role in intracellular pH homeostasis in context with histidine metabolism.

### Membrane potential is altered by mutations in EriC or EriC2

**Fig. 6:**
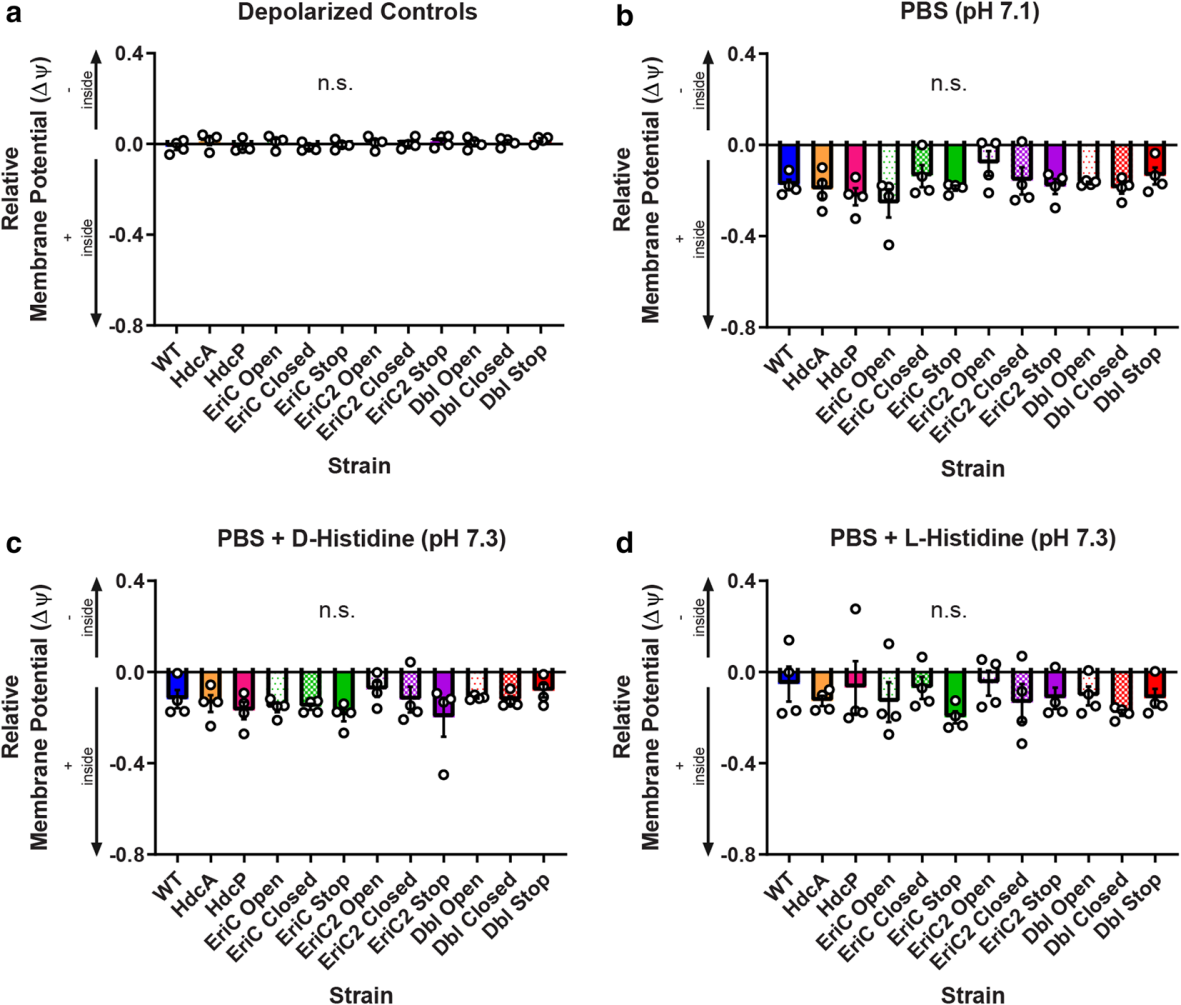
Membrane potential measurements for WT and mutant *L. reuteri* strains under neutral conditions. 24-hour MRS cultures were washed and loaded with DiOC_2_ [3], and resuspended in PBS alone (**b**), +CCCP (**a**), +15 mM d-histidine (**c**), or +15 mM l-histidine (**d**). Signal was recorded in red and green channels in a plate fluorescence reader. N = 4 biological replicates per group. Two-way ANOVA with Tukey’s multiple comparison test. Error bars represent ± SEM. Under neutral conditions, there were no significant differences (n.s.) in membrane potential among treatments or across strains. *n.s.* not significant

**Fig. 7:**
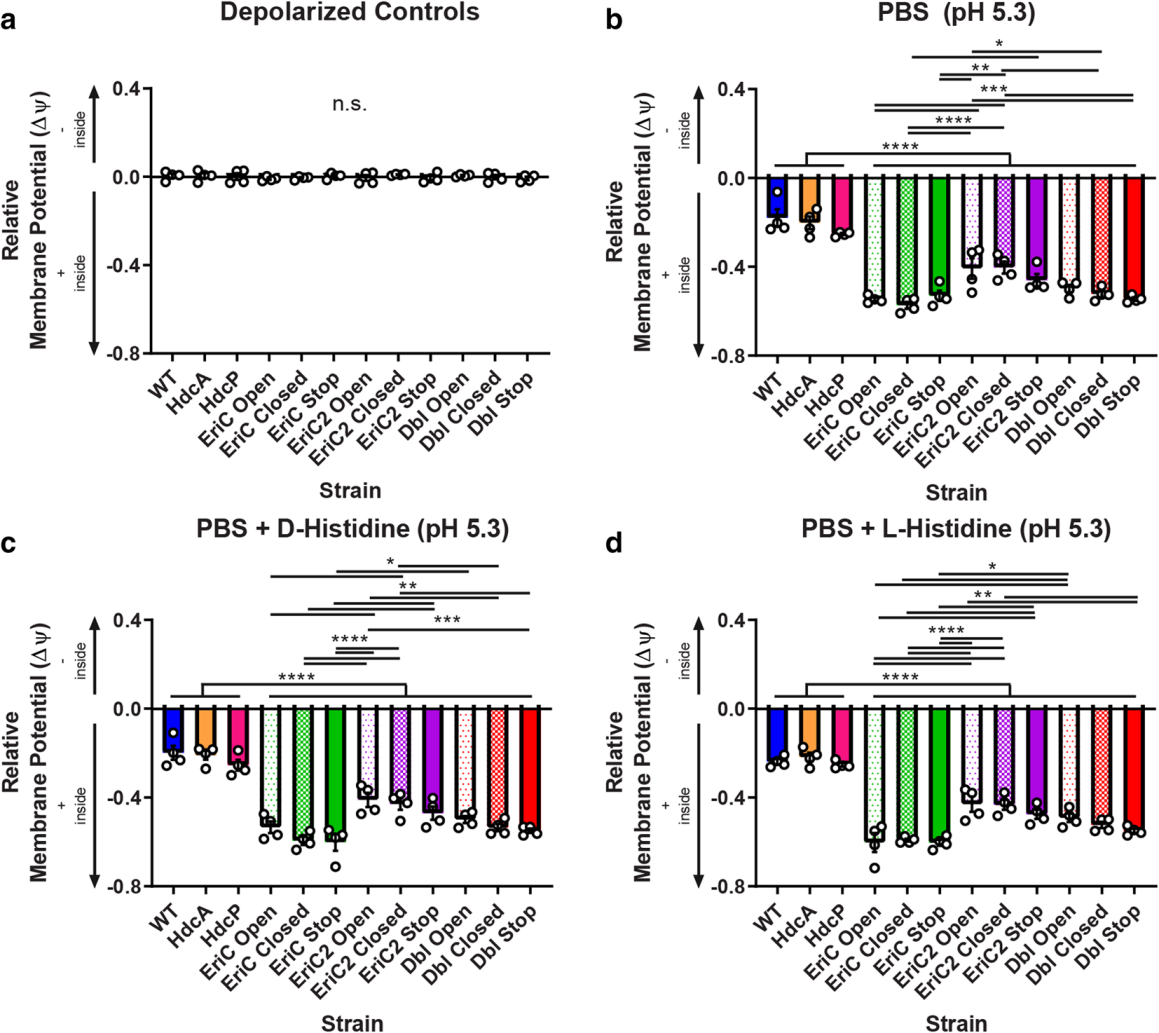
Membrane potential measurements for WT and mutant *L. reuteri* strains under acidic conditions. *L. reuteri* cells were treated as in Fig. 6, except the pH of assay buffers was lowered to 5.3. N = 4 biological replicates per group. Two-way ANOVA with Tukey’s multiple comparison test. Error bars represent ± SEM. Under acidic conditions, all EriC and EriC2 mutant strains exhibited a significantly positive polarization relative to WT, HdcA Stop, and HdcP stop cells. Additionally, within the PBS, d-His, and l-His treatment groups, significant differences were observed for the strains marked above (*P < 0.05, **P < 0.01, ***P < 0.001, ****P < 0.0001, *n.s.* not significant)

To finish our examination of the interactions between HDC components and ClC-family transport activity, we measured membrane potential in wild type and mutant *L. reuteri* using the potentiometric dye, 3,3′-diethyloxacarbocyanine iodide (DiOC_2_ [3]). Bacteria were cultured under standard conditions, stained briefly with DiOC_2_ [3] in PBS, and resuspended in neutral (pH 7.1–7.3, Fig. 6) or acidic (pH 5.3, Fig. 7) PBS, either alone, or with a 15 mM d-, or l-histidine supplement. Samples of each strain were also depolarized with carbonyl cyanide *m*-chlorophenyl hydrazone (CCCP) as controls. The dye used in this experiment emits stable green fluorescence and potential-sensitive red fluorescence. The relative membrane potential (Δψ) for each group is reported as the baseline-normalized red/green fluorescence ratio, and analyzed using two-way ANOVA with Tukey’s multiple comparison test (N = 4 per group). For both neutral and acidic conditions, control CCCP-treated cells have an approximate normalized red/green ratio of 0 (Figs. 6a and 7a). Under neutral conditions, no significant differences were observed among strains or treatments, although a tendency towards positive polarization of the membrane relative to the CCCP controls was observed across all treatment groups and strains (Fig. 6b–d). Under acidic conditions, membrane potential became significantly more positive relative to CCCP controls for all treatments of EriC, EriC2, and double mutant strains (Fig. 7b–d, *-****P < 0.05-0.0001). These data support the model proposed for *E. coli* [38], in which the bacteria may actually maintain an internally-positive membrane potential under acidic conditions, and ClC transporters serve to balance this charge difference through the import of chloride ions.

## Discussion

In this study, we have shown that extracellular pH and ion (Cl^−^) gradients can affect the synthesis of one of *L. reuteri*’s important immunomodulatory molecules, histamine. Moreover, histamine synthesis by this strain can be affected by the activity of two ClC proton/chloride antiporters (EriC and EriC2). We provide evidence that expression and activities of these transporters are differentially controlled. Despite these differences, the EriC and EriC2 transporters can modulate the intracellular pH and membrane potential of *L. reuteri*, and likely impact microbe:host communication. By understanding how these systems are functioning in bacterial cells, we can predict how metabolite-generating processes such as amino acid decarboxylation by *L. reuteri* and other microbes may respond to environmental changes within the mammalian intestine.

We have shown that the transport activity of ClC-family proton/chloride antiporters can profoundly impact *L. reuteri*’s histidine decarboxylase system despite not playing a direct role in the import, conversion, or export of histidine/histamine. This conclusion is evidenced by the significantly decreased histamine output from bacterial strains with genetically modified ClC transporters. Although these antiporters have been studied in conjunction with other amino acid decarboxylase systems [30, 31, 38, 39], this report includes the first study to examine their functional roles in the histidine decarboxylase system.

Several amino acid metabolism pathways contribute to acid resistance in bacteria. In addition to histidine, aspartic acid, glutamate, glutamine, and arginine can all be metabolized in proton-consuming reactions [4, 31, 39, 40]. Some mechanisms (like deamidation of glutamine to glutamate or arginine to ornithine) are conserved among bacteria, and can be found in many species [40, 41], while others (glutamate or histidine decarboxylation, urea hydrolysis), are strain-dependent systems. *L. reuteri* 6475 for example, does not contain a glutamate decarboxylase system or a urease system, even though these pathways are found in other strains of *L. reuteri* (per IMG database [42]). Despite lacking these other common acid resistance systems, our data suggest that the histidine decarboxylase system is not essential for the survival of *L. reuteri* under acid stress. However, histamine-generating capacity is known to be an important feature of *L. reuteri’*s probiotic effect [12, 14, 17, 25].

We have demonstrated that even single amino acid substitutions in either ClC transporter are sufficient to reduce histamine output, but the degree of this effect varies both by mutation state and by the affected gene. In either gene, we would predict that the “Open” conformation would allow constitutive gradient-dependent movement of chloride ions, which could result in altered intracellular chloride concentrations. Previous work studying the glutamate decarboxylase systems in *E. coli* and *Lactococcus lactis*. have identified cis- and trans- activation elements that respond to chloride, and similar mechanisms might also regulate HDC. In *E. coli*, the glutamate decarboxylase GadB itself has allosteric sites for chloride binding, that increase the rate of decarboxylation [43]. However, to our knowledge, no chloride binding sites have been documented for glutamate decarboxylases (GadB) or histidine decarboxylases (HdcA) in *Lactobacillus*. Despite their similar functional roles, GadB and HdcA belong to mechanistically distinct enzyme classes. GadB is a pyridoxal 5′-phosphate dependent decarboxylase, and HdcA is a pyruvate-dependent decarboxylase. As such, they are unlikely to share allosteric regulatory mechanisms [18, 43, 44]. In *L. lactis*, a chloride-responsive promoter was found to increase expression of the entire glutamate decarboxylase gene cluster via a response regulator, GadR [45]. GadR has also been found in some (but not all) *Lactobacillus* species that possess glutamate decarboxylases, but its chloride sensitivity has not been assessed in this genus [46, 47]. When EriC transport is disrupted, we observed a two-fold decrease in *hdcA* expression (suggesting a decrease in intracellular chloride, if regulated by this mechanism). A chloride-responsive promoter seems plausible in this system, but no such promoters have been identified in *L. reuteri.* A transregulator of HdcA expression, RsiR (*L. reuteri*- specific immunoregulator), was found to activate the putative promoter region upstream of the HDC, but this factor was not assessed for chloride-responsivity [19].

Previous studies have suggested that pH and chloride content may affect expression and activity of pyruvoyl-dependent HDCs in lactic acid bacteria [4, 18, 24, 33, 48, 49]. In general, acidic pH increases activity of the HdcA enzyme (maximum activity near pH 4), and *hdcA* expression is induced at terminal growth phases (which coincide with acidic pH in batch culture of lactic acid bacteria), although these effects may vary by species [18, 24, 33, 48]. In *S. thermophilus,* the addition of up to 5% w/v (855 mM) NaCl to the growth medium trended toward increased histamine output [33]. Other factors such as presence of supplementary histidine can affect the degree of *hdcA* induction [19, 48]. Our data indicate that alterations in EriC (leading to more acidic intracellular pH, and more positive membrane potential) decreased the expression of *hdcA*. This potentially compensates for the fact that we would have expected increased histamine output given the lower intracellular pH (i.e. less *hdcA* is expressed, but the available HdcA enzyme is more active). This study also provides evidence regarding the regulation of the expression of EriC and EriC2. Both EriC and EriC2 have reduced expression when they are functionally inactivated. However, we cannot determine in this study if this is indicative of a regulatory feedback loop, or if the mutant-transcript is more susceptible to RNA decay.

*E. coli* generally maintain their intracellular pH near neutral, and several mechanisms function to alkalinize the interior in response to acid stress [38]. However, our data show that wild type *L. reuteri* maintain an intracellular pH in excess of 1 unit lower than a neutral external environment. Our data indicate that altering the EriC2 transporter allows for a more alkaline interior compared to wild type *L. reuteri*. As predicted given that histidine decarboxylase activity decreases with alkalinity, these strains have the lowest histamine output among the ClC mutants. Conversely, strains with mutations in EriC have more acidic interiors than the wild type strain. These strains have commensurate increases in histamine output compared to the EriC2 mutants, but still produce less histamine than wild type strains, indicating likely inhibition of histidine/histamine exchange. Although numerical differences in pH between the wild type and mutant strains, we remind the reader that for every 0.3 unit increase in pH, the intracellular concentration of protons is cut in half. Thus, even a small flux in intracellular pH can have a significant impact on biological reactions occurring within the cell [50]. Given that mutations in EriC tended to result in a more acidic intracellular pH, and mutations in EriC2 tended to have a more alkaline intracellular pH, we might predict the double mutants to have a null effect. But they display intracellular pH patterns closer to EriC2, indicating that EriC2 may be the dominant controller of pH in this system. The ability of ClC transporters to modulate intracellular pH has important implications for protein synthesis in industrial or therapeutic applications. Intracellular fractions of exogenously expressed proteins can often have reduced activity because they are unsuited for the intracellular pH of the producing organism [51]. Modulating intracellular pH by manipulation of ClC transport may help improve active enzyme yields.

In our membrane potential study, *L. reuteri* was found to have a membrane potential that was more positive than depolarized control cells. The degree of this relatively positive membrane potential is more pronounced in the EriC strains compared to the EriC2 strains, indicating that the EriC transporter may be the dominant controller of membrane potential in this system. Internally-positive membrane potentials have also been observed in other species of bacteria, particularly among those that thrive in acidic environments. In *Thiobacillus acidophilus*, a positive Δψ as high as + 140 mV was measured under extreme acid stress (pH 1.0), and fell to nearly + 0 mV as pH approached neutral. The authors noted that this voltage change was not met with a commensurate change in intracellular pH, indicating either expulsion of anions or intake of additional non-proton cations [52, 53]. Potassium is a likely candidate for this cation, as high intracellular concentrations of K^+^ have been measured in *T. acidophilus* (~ 400 mM), and have been measured as high as 650 mM in *Bacillus* spp [52–54]. Future studies measuring intracellular potassium concentration in *L. reuteri* may help determine how it is able to maintain its membrane potential in acidic environments.

**Fig. 8:**
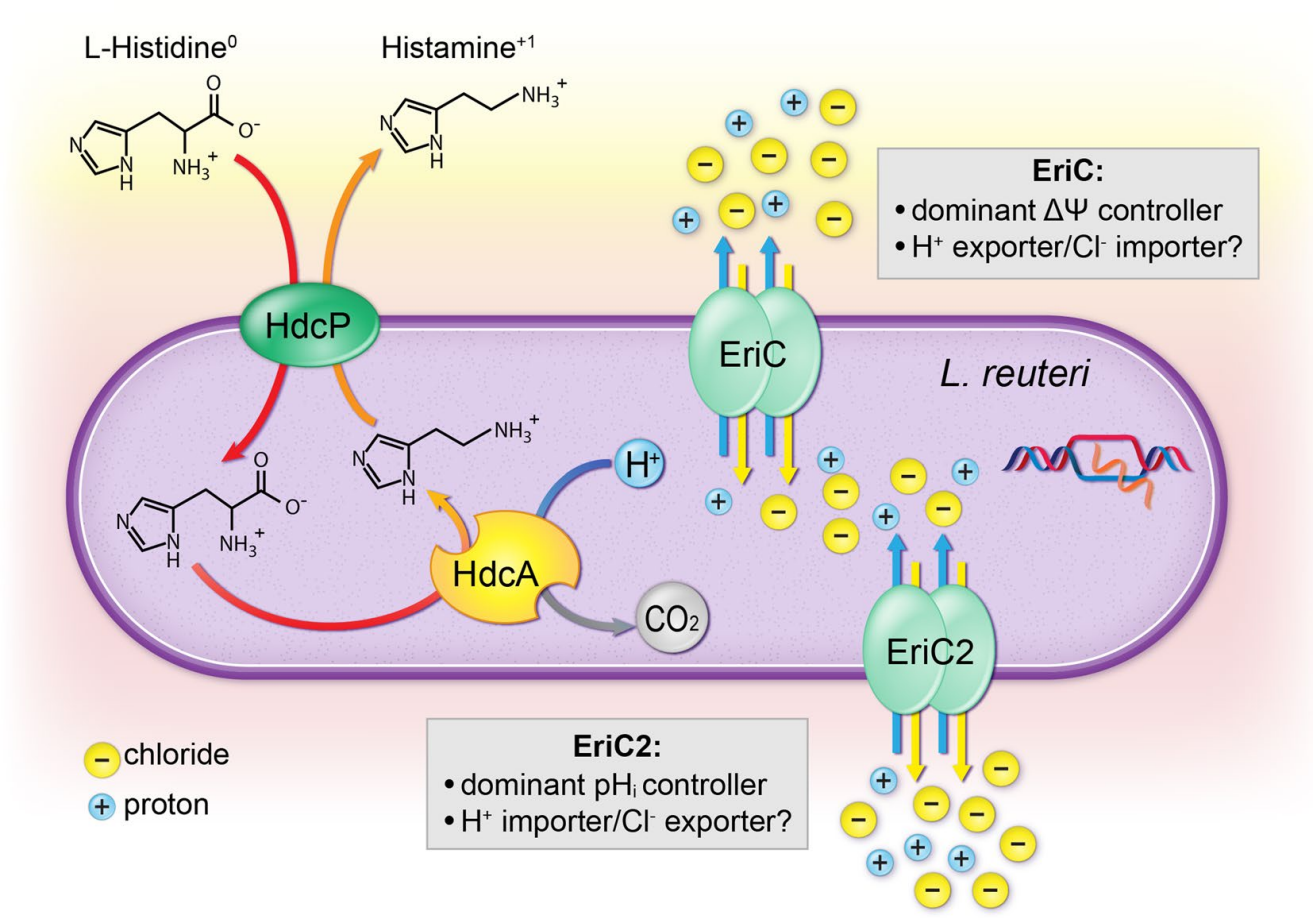
Proposed model of interactions among *L. reuteri’*s HDC proteins and ClC transporters. Phenotypic data suggest that EriC dominates over EriC2 in control of membrane potential, while EriC2 seems to play a greater role than its counterpart in controlling intracellular pH. EriC and EriC2 appear to use opposing transport mechanisms with EriC being a proton exporter/chloride importer while EriC2 exhibits the behavior of a proton importer/chloride exporter. Despite differing transport mechanisms, both play a role in maintaining electrochemical balance within the cell during histamine production and as controllers of intracellular pH in this system

Two competing models provide different perspectives regarding how ClC transporters behave in concert with decarboxylase systems. One model suggests that ClC transporters act as electrochemical shunts, balancing strong internally-negative membrane potential that may occur due to the outward movement of protons following decarboxylation of amino acids (arginine or glutamate, in *E. coli*). In these studies, deletion of ClC transporters yielded reduced intake of glutamate and arginine, as well as subsequently reduced output of amino acid decarboxylation products (GABA and agmatine) while under acid stress. Using liposome studies with recombinant ClcA, they also demonstrated that chloride export could be dramatically increased upon exposure to extracellular acidity [28, 30]. A second group demonstrated that providing glutamate or arginine to *E. coli* under acid stress helped to increase the intracellular pH. Functional decarboxylase systems enabled bacteria to reverse their normally inside-negative membrane potential (− 50 mV at stationary phase) to an inside-positive + 30 to + 80 mV [31]. ClC transporters might act as chloride importers, serving to balance the internally-positive potential at these membranes [31, 38]. Neither group worked with a species with two naturally occurring, yet genetically-distinct ClC transporters Given that ClC proton/chloride antiporters are capable of gradient-dependent movement of either ion, both models are theoretically possible [28, 29]. Our histamine synthesis data demonstrate that both the “Open” and “Closed” mutation state in each gene can reduced the amount of histamine produced. Given that histamine synthesis might be impacted by a change in pH or membrane potential, this result is not surprising. Intriguingly, mutations within a gene (e.g. EriC vs. EriC2) produce more similar effects on the physiological parameters of pH and membrane potential than the mutation state (“Open” vs. “Closed”). Since ClC transporters have been demonstrated to conduct proton/chloride antiport in either direction, we propose that EriC and EriC2 might each be committed to one direction of antiport (with EriC being a chloride importer/proton exporter, and EriC2 being a chloride exporter/proton importer) (Fig. 8).

Our work demonstrates that the external ion environment can disrupt the synthesis of a particular metabolite by a single bacterium, but other recent studies have demonstrated the importance of the external ionic environments in shaping the structure and function the intestinal microbiota. For example, loss or inhibition of the mammalian Na^+^/H^+^ exchanger NHE3 significantly altered luminal pH and anion content intestines of mice, which in turn altered the Bacteroidetes/Firmicutes ratio, and conferred a growth advantage to *Clostridioides difficile* [55, 56]. In human and mouse studies, other groups have also shown significant changes in composition and metabolic function of the intestinal microbiota in the absence or inactivation of the Cystic Fibrosis Transmembrane Conductance Regulator (CFTR) chloride channel [57–59].

A survey of the Human Microbiome Project’s gastrointestinal reference genome set reveals that approximately 50 strains from 21 genera have histidine decarboxylases, and thus may be capable of producing histamine [42]. However, only a small fraction of these strains have actually been assessed for histamine secretion [7, 18]. As natural and engineered probiotics become more commonplace, we will need to understand the genetic and environmental regulatory mechanisms governing production of bioactive compounds like histamine. ClC transporters are attractive targets in modulating the production of these molecules. In situations where bioactive amines may act as contaminants, disabling ClC transport could help limit their synthesis [60]. When used as probiotics, biogenic amine synthesis may be promoted by increasing ClC activity through increased gene expression or protein function [14, 61].

## Conclusion

The data presented here suggest that the expression and activity of *L. reuteri’s* EriC and EriC2 antiporters may be governed by different physiological states despite their high degree of structural similarity. The expression of *eriC* and *eriC2* appear to be differentially regulated, and mutations in each gene can yield different effects on the expression of the *hdcA* gene encoding histidine decarboxylase. Transport-specific and functionally-inactivating mutations in EriC and EriC2 generate opposing intracellular pH phenotypes, which may indicate that each transporter fulfills a different biological role in *L. reuteri* (Fig. 8). When both transporters are inactivated, we do not observe the synergistic antagonism that we might predict from functionally related proteins. This finding may indicate that other mechanisms, such as chloride or proton channels of different families or central carbon metabolism and activation of ATPases, may compensate for ClC transporter activity in their absence [4]. Taken together, our data indicate that *L. reuteri*’s ClC transporters play important roles in the histidine decarboxylase system, and each transporter may have a different net physiological effect on the microbial cell. Combined with the growing body of the molecular knowledge of ClC transporter activity, this work provides new avenues for studying how proton/chloride antiport may function *in vivo* to impact microbial metabolism [29, 62].

## Materials and methods

### Strains and culturing conditions

*Lactobacillus reuteri* ATCC PTA 6475 was a kind gift of BioGaia AB. For all assays, strains (Additional file 3: Table S1) were first streaked on de Man-Rogosa-Sharpe (MRS) agar (Becton Dickinson). Single colonies were inoculated into MRS broth and cultured overnight (14–18 h) anaerobically at 37 °C (AS-580 workstation, Anaerobe Systems, 5% CO_2_, 5% H_2_, and 90% N_2_ gas atmosphere). Optical density at 600 nm (OD_600_) was measured on a BioRad SmartSpec 3000 spectrophotometer. Cultures were diluted to an OD_600_ of 0.1 in fresh MRS and grown as described above for the time indicated for each experiment. As needed, 10 µg/mL erythromycin was added to liquid or solid media for plasmid maintenance.

### Histamine production assays

Single colonies of wild type or mutant *L. reuteri* were inoculated into 200 µL MRS broth in a non-tissue culture treated 96-well plate (VWR International) and grown overnight under standard conditions. Optical density was measured in a Synergy HT multimode plate reader (Biotek) with path length correction, and samples were diluted in fresh MRS to OD_600_ = 0.1 and grown for 24 h. OD_600_ was measured and cells were then diluted to OD_600_ = 1.0. Two hundred µL were transferred to a 0.22 µm pore PVDF filter plate (Millipore #MSGVS2210), and filtered by vacuum. Cells were washed twice with 200 µL PBS, then resuspended in an assay-specific buffer containing l-histidine. The plate was sealed with plastic film to prevent evaporation and incubated under standard conditions for 3.5 h. Buffer was then sterilized and collected by vacuum filtration and stored at − 20 °C until analysis by liquid chromatography-mass spectrometry (LC–MS). The histamine production assay solution consisted of 98.6 mM dibasic potassium phosphate and 50.7 mM citric acid with 15 mM l-histidine and 80 mM sodium chloride. Buffer compositions for the variable pH and variable chloride histamine production assays are described in Additional file 3: Tables S2A and B. One-way ANOVA with a Tukey’s post-test was used to assess differences in normalized histamine output among treatment conditions (N = 4 per strain).

### Histamine quantification by LC–MS

Histamine quantification was performed by A. Haag and colleagues at the Texas Children’s Microbiome Center (TCMC). Histamine, formic acid (FA), and perfluoroheptanoic acid (PFHA) were obtained from Sigma Aldrich (St. Louis, MO). Histamine-α,α,β,β-d4 was obtained from CDN Isotopes (Point-Claire, Canada). Water and acetonitrile (ACN) were obtained from Thermo-Fischer Scientific (Waltham, MA). The internal standard was prepared at a concentration of 100 ng/mL histamine-d4 in water.

To prepare samples for LC–MS analysis, frozen samples were thawed and immediately vortexed for 1 min. The samples were then centrifuged at 10,000 rpm for 5 min. Ninety µL of internal standard was added to 10 µL of each sample and vortexed. Samples were then loaded into 0.5 mL autosampler vials for quantification.

Chromatography was performed on a Shimadzu (Kyoto, Japan) Nexera-XR HPLC system consisting of an SIL-30ACMP autosampler, a CTO-20AC column oven and 2 LC-20ADxr binary pumps. Five µL of sample were loaded onto a Phenomenex (Torrance, CA) 1 mm × 50 mm Luna phenylhexyl reversed phased column equipped with a Phenomenex Luna phenylhexyl 4 mm x 2 mm guard column. The aqueous mobile phase (A) consisted of H_2_O:ACN:FA:PFHA (99.3:0.5:0.1:0.1 v/v/v/v) and the organic mobile phase (B) consisted of H_2_O:FA (99.9:0.1 v/v). Column flow was 80 µL/min. The elution gradient was optimized as follows: Started from 20% B and increased to 80% B over 6 min; held for 1 min; ramp back to 20% B over 6 s and maintained at 20% for a total chromatographic run time of 12 min.

Selected reaction monitoring was performed on a Sciex (Framingham, MA) 6500 QTRAP with a Turbo V source. The mass spectrometer was operated in the positive ion mode under the following conditions: curtain gas: 20 psi; collision gas: HIGH; spray voltage: 4.5 kV; ion source gas 1: 20 psi; ion source gas 2: 20 psi; interface heater temperature 175 °C; Q1 and Q3 resolution: unit; scan time: 100 ms; de-clustering potential: 100 V; entrance potential: 8 V; collision exit potential: 10 V. The instrument was calibrated using Sciex PPG calibration standard and tuned to the manufacturer’s specifications. SRM transitions monitored for histamine were 112.1 → 95.1 (20 eV) and 112.1 → 68.1 (30 eV). Data shown in the figures herein correspond to the 112.1→95.1 transition. For histamine-d4, the SRM transitions 116.1 → 99.1 (20 eV) and 116.1 → 72.1 (30 eV) were monitored. Data were acquired with Analyst Software^®^ (ver 1.6.2).

Quantification was performed with Multiquant™ Software (ver 3.0.1) using the following parameters: Gaussian smooth width: 3 points; RT half window: 30 s; minimum peak width: 3 points; minimum peak height: 1000; noise percentage: 40%; regression fit: linear; regression weighting: 1/x. An R^2^ = 0.999 or better was required and a minimum 6-point calibration was used. Raw histamine values were normalized to the OD_600_ values of the cultures used in the assay. One-way ANOVA with a Dunnett’s post-test was used to assess differences in normalized histamine output between mutant strains and the wild type (N = 4–6 per strain).

### Identification of proton/chloride transporters and their gating glutamate residues in *L. reuteri*

Genome sequences for *L. reuteri* ATCC PTA 6475 (MM4-1a), *Escherichia coli* K-12 MG1655, *Mus musculus* C57BL/6, and *Homo sapiens* were obtained from the Integrated Microbial Genomes (IMG) Database (Additional file 3: Table S3) [42]. Each genome was searched for the functional annotation COG0038 (Clusters of Orthologous Groups 0038: H^+^/Cl^−^ antiporter ClcA). The resulting genes were aligned using IMG’s ClustalOmega algorithm. Alignments were searched manually for the presence of the internal and external gating domains shared by proton/chloride transporters, but not by related voltage gated chloride channels. Previous work in *E. coli* has identified individual glutamate residues within each of these domains that are absolutely essential for coupled proton/chloride exchange. Antiporters have the conserved glutamate residue in both domains, while the closely related channels uniformly lose the glutatmate in the internal gating site [28, 34, 35]. To demonstrate inter-kingdom consensus in these highly conserved domains, sequence logos were generated for the region surrounding these glutamate residues using WebLogo [63]. To further demonstrate the similarity between *L. reuteri*’s proton/chloride antiporters, and the one found in *E. coli*, we generated in silico 3D structural models using SwissModel [64]. For each protein, the 3.5 Å X-ray crystal structure of the ligand-free homodimer of *E. coli* ClcA was used as a template (PDB: 1kpk). Models were visualized using MOLMOL (v2K.2) [65].

### Bacterial mutagenesis

RecT-mediated single stranded DNA recombineering was used as described previously to introduce specific amino acid changes into the coding sequence of *eriC* and *eriC2* [66]. Briefly, cultures were grown in MRS to an OD_600_ of 0.45–0.55. Expression of the RecT single-stranded DNA binding protein was induced from a plasmid (pJP042, a gift of R. Britton). Cells were then washed twice in 0.5 M sucrose/10% glycerol for electrocompetence. Ninety-bp single-stranded DNA consisting of the mutant codon flanked by homologous sequences (Additional file 3: Table S4) were then electroporated into cells in a single pulse using a BioRad GenePulser at 2500 kV, 25 µF capacitance and 400 Ω resistance. Following recovery in MRS, mutants were purified by two successive rounds of screening by mismatch amplification mutation assay (MAMA)-PCR (Additional file 3: Table S5). The recombineering plasmid was purged via passage without antibiotics until susceptibility was achieved. Mutations were verified by Sanger sequencing (Lone Star Labs, Houston, TX) prior to stock generation and additional experiments. Growth parameters, including culture density (OD_600_), and viability as determined by plating and counting colony forming units (CFU) per mL were determined for each mutant strain compared to WT (Additional file 1: Figure S1). Two-way repeated measures ANOVA with Dunnett’s multiple comparison test was used to detect differences in growth dynamics relative to wild type (N = 3 per strain per timepoint).

### Expression analysis

*Wild type* and mutant *L. reuteri* were cultured under standard conditions for 24 h. One mL of each culture was fixed by addition of 1 mL ice-cold methanol and pelleted by centrifugation at 16,000×*g* for 30 s. Supernatants were discarded, and pellets were placed on ice. RNA extraction was performed using the Zymo QuickRNA kit with slight modification. Cells were first resuspended in 100 µL STE buffer (100 mM NaCl, 10 mM Tris-HCl, pH 8.0, 1 mM EDTA), and transferred to 1.5 mL screw-top tubes containing ~ 100 µL 0.1 mm glass beads. Samples were processed on a FastPrep bead homogenizer (MP Biologicals) for 20 s at 4.0 m/s. Zymo Quick RNA lysis buffer was added to each tube, and samples were homogenized again. Debris was settled by brief centrifugation at 10,000×*g*, and RNA was obtained from the supernatant according to the manufacturer’s protocol. Following extraction, further genomic DNA elimination was performed using the Ambion Turbo DNA*Free* kit. Concentration and RNA quality (260/280 nm and 260/230 nm ratios) was assessed by Nanodrop (ThermoScientific). One µg of cDNA was generated from RNA using the Bioline SensiFast cDNA synthesis kit. cDNA was diluted 1:4 in nuclease-free water and 4 µL was used per qPCR reaction. Primers (Additional file 3: Table S5) were designed for genes of interest using Primer3 [67], and validated for single products using *wild type L. reuteri* 6475 genomic DNA. Each qPCR was performed on a QuantStudio3 qPCR machine (Applied Biosystems) in 20 µL reactions using Fast SYBR green master mix (Applied Biosystems) and 40 nM each forward and reverse primers (Integrated DNA Technologies). Fold changes in cycle threshold (Ct) were determined between genes of interest and the housekeeping gene *rpoB* (RNA polymerase, β subunit) using the 2^−ΔΔCt^ method [36]. For each gene, the average difference in Ct between the gene of interest and *rpoB* for the *wild type* strain was used as the control Ct for expression in the mutant strains. One-way ANOVA with a Dunnett’s post-test was used to assess differences between mutant strains and the *wild type* (N = 6 biological replicates).

### Intracellular pH assay

Cultures were grown in MRS for 24 h from an OD_600_ = 0.1 in 96-well plates as in the histamine production assay. Fifty µL of each culture were transferred to a conical bottomed 96-well plate and pelleted via centrifugation at 2000×*g* for 5 min. Supernatants were discarded and cells were washed twice in live cell imaging solution (LCIS, Molecular Probes). Cell pellets were then resuspended in LCIS containing 1× pHrodo Red AM dye (provided as 1000× in dimethyl sulfoxide, DMSO) and 1× PowerLoad (provided as 100×) (Molecular Probes), and incubated at 37 °C on an orbital shaker at 220 rpm for 30 min. Following incubation, cells were pelleted again to remove excess staining solution, resuspended in 100 µL LCIS, and immobilized on a 0.22 µm-pore PVDF filter plate (Millipore) via a vacuum manifold with ~ 5 inHg suction. Filters were washed once by vacuum and wells were refilled with LCIS. The filter plate was then loaded into a Synergy HT plate reader with incubation at 37 °C. A citrate buffer series was used to examine intracellular pH due to the wide pH range that can be covered without addition of HCl and its previous successful use in profiling intracellular pH in *L. reuteri* strains [40, 68]. In addition, *L. reuteri* 6475 is not predicted to have the enzymes necessary to utilize citrate as a carbon source for metabolism [69]. Fluorescence at an excitation wavelength of 560 nm and an emission wavelength of 590 nm was recorded every 5 min over the following sequential buffer conditions (Additional file 3: Table S6): [1] 5 min in LCIS, [2] 20 min in potassium citrate, pH 5.0 with 15 mM d-histidine, [3] 20 min in potassium citrate, pH 5.0 with 15 mM l-histidine. Cells were washed twice with LCIS prior to generating standard curves of fluorescence versus intracellular pH for each cell population. To generate standard curves, fluorescence readings were taken for 10 min each at 5 min intervals in potassium citrate buffers at pH 4.5, 5.5, 6.5, and 7.5 in the presence of 10 μM valinomycin and 10 μM nigericin to equilibrate intra- and extracellular pH. Linear regression lines were fit to the standard curve of each sample (Microsoft Excel) and used to calculate the intracellular pH during the experimental readings. Each data point in the resulting fluorescence traces represents several replicates (N = 12) per bacterial strain. Two-way repeated measures ANOVA analysis with Dunnett’s multiple comparison test was used to determine statistical differences between strains. A pilot experiment (N = 6) was performed as above with *wild type L. reuteri* co-stained with pHrodo Red as described above and Hoechst 33342 DNA stain (10 μg/mL final concentration, Ex 353 nm/Em 483 nm) to ensure minimal loss of whole cell signal during the vacuum plate assay (Additional file 2: Figure S2). Representative images of stained *wild type L. reuteri* under native and standard curve conditions were taken on a Zeiss AxioImager Z1 microscope with a Hamamatsu Electron Multiplier CCD camera. Overlapping phase contrast and Texas Red fluorescence layers were acquired for each condition with a 100x oil immersion lens using the same exposure and contrast settings for each sample.

### Membrane potential assay

Membrane potential (Δψ) measurements were determined for *L. reuteri* strains using the fluorescent potentiometric dye 3,3′-diethyloxacarbocyanine iodide (DiOC_2_ [3], ThermoFisher) [70, 71]. Cells were cultured for 24 h as described previously. For each strain, 8 × 100 µl aliquots were transferred to a conical-bottomed 96-well plate and pelleted by centrifugation at 2000×*g* for 2 min. Pellets were resuspended in potassium-free PBS (7 mM Na_2_HPO_4_, 3 mM NaH_2_PO_4_, 140 mM NaCl) containing 3 µM DiOC_2_ [3] (provided as 1000× in DMSO). Cells were incubated in staining solution at room temperature for 5 min, protected from light. Stained cells were then pelleted and resuspended in a test buffer (PBS, at pH 7 or pH 5, with or without 15 mM l- or d-histidine) (Additional file 3: Table S7). One sample of each strain per experiment was depolarized by resuspension in PBS with 2.5 µM carbonyl cyanide 3-chlorophenylhydrazone (CCCP). Stained cells in buffer were transferred to a black-walled, clear-bottomed 96 well plate. Fluorescence was measured on a Synergy HT multimode plate reader with emission/excitation wavelengths of 488 nm/528 nm (green) and 488 nm/635 nm (red). For each strain in each experiment, gain values on the reader were adjusted such that green and red fluorescence values were approximately equal for the depolarized sample (red/green ratio of depolarized control samples set to ~ 1.0). A qualitative value for membrane potential was determined from the ratio of red fluorescence to green fluorescence in each well minus 1.0. A positive red/green ratio (> 0) indicates negative polarization relative to control cells, while a negative ratio (< 0) suggests a positive polarization. A two-way ANOVA with Tukey’s multiple comparison test was used to determine deviations from the *wild type* potential.

### Statistical analyses

Experiment-specific tests are described in their respective methods sections. All analyses were performed with GraphPad Prism v. 8.0.1. All data are presented as averages ± SEM (standard error of the mean).

## Supplementary information

**Additional file 1: Figure S1.** Growth parameters of *L reuteri* wild type 6475 and mutant strains in MRS medium over 48 h. Optical density (A) and viability (B) of bacterial cultures were measured at regular intervals during lag, exponential, and stationary phases. N = 3 per strain. Error bars represent ± SEM. Stars represent maximum P-values from a two-way repeated measures ANOVA with Dunnett’s multiple comparison tests performed within each timepoint relative to WT.

**Additional file 2: Figure S2.** Comparison of pHrodo and Hoechst signals during intracellular pH assay procedure. Red trace indicates pHrodo fluorescence and blue trace indicates Hoechst fluorescence during a pilot intracellular pH assay. N = 6. Error bars represent ± SEM. The stability of the Hoechst signal throughout the assay suggest signal is not being lost due to cell lysis throughout the testing period.

**Additional file 3: Table S1.** Description of bacterial strains used in this study. **Table S2. A** Buffer composition: variable pH histamine production assay (Fig. 1a). **B** Buffer composition: variable Cl^−^ histamine production assay (Fig. 1b)**. Table S3.** Description of genes and genomes used in *in silico* alignments in this study. **Table S4.** DNA oligonucleotides used in this study for mutagenesis. **Table S5.** Primers used in this study for screening and quantitative PCR. **Table S6.** Composition of non-commercial buffers used in the intracellular pH assay. **Table S7.** Composition of non-commercial buffers used in the membrane potential assay.

## Data Availability

All data generated or analyzed during this study are included in this published article

## Abbreviations

ACN: acetonitrile
AM: acetoxymethyl ester
ANOVA: analysis of variance
ATCC: American Type Culture Collection
CCCP: carbonyl cyanide *m*-chlorophenyl hydrazone
CCD: charge-coupled device
cDNA: complementary DNA
CFU: colony-forming unit
ClC: chloride channel family
COG: clusters of orthologous groups
Dbl: EriC/EriC2 double mutant strain
ΔΨ: membrane potential
DiOC_2_(3): 3,3′-diethyloxacarbocyanine iodide
FA: formic acid
HDC: histidine decarboxylase gene cluster
IMG: integrated microbial genomes database
inHg: inches of mercury (unit of vacuum pressure)
LCIS: live cell imaging solution
MAMA-PCR: mismatch amplification mutation assay-PCR
MRS: de Man-Rogosa-Sharpe bacterial medium
OD_600_: optical density at 600 nm
PDB: protein data bank
PFHA: perfluoroheptanoic acid
PPG: propylene glycol polymers
PVDF: polyvinylidene fluoride
qPCR: quantitative PCR
SEM: standard error of the mean
SRM: selected reaction monitoring
STE: Sodium-Tris-EDTA buffer
WT: wild type
