# Supplementary figures and images for: ClC transporter activity modulates histidine catabolism in Lactobacillus reuteri by altering intracellular pH and membrane potential
Source: Microb Cell Fact. 2019 Dec 12;18:212. doi: 10.1186/s12934-019-1264-0 (PMC6909576; doi:10.1186/s12934-019-1264-0)

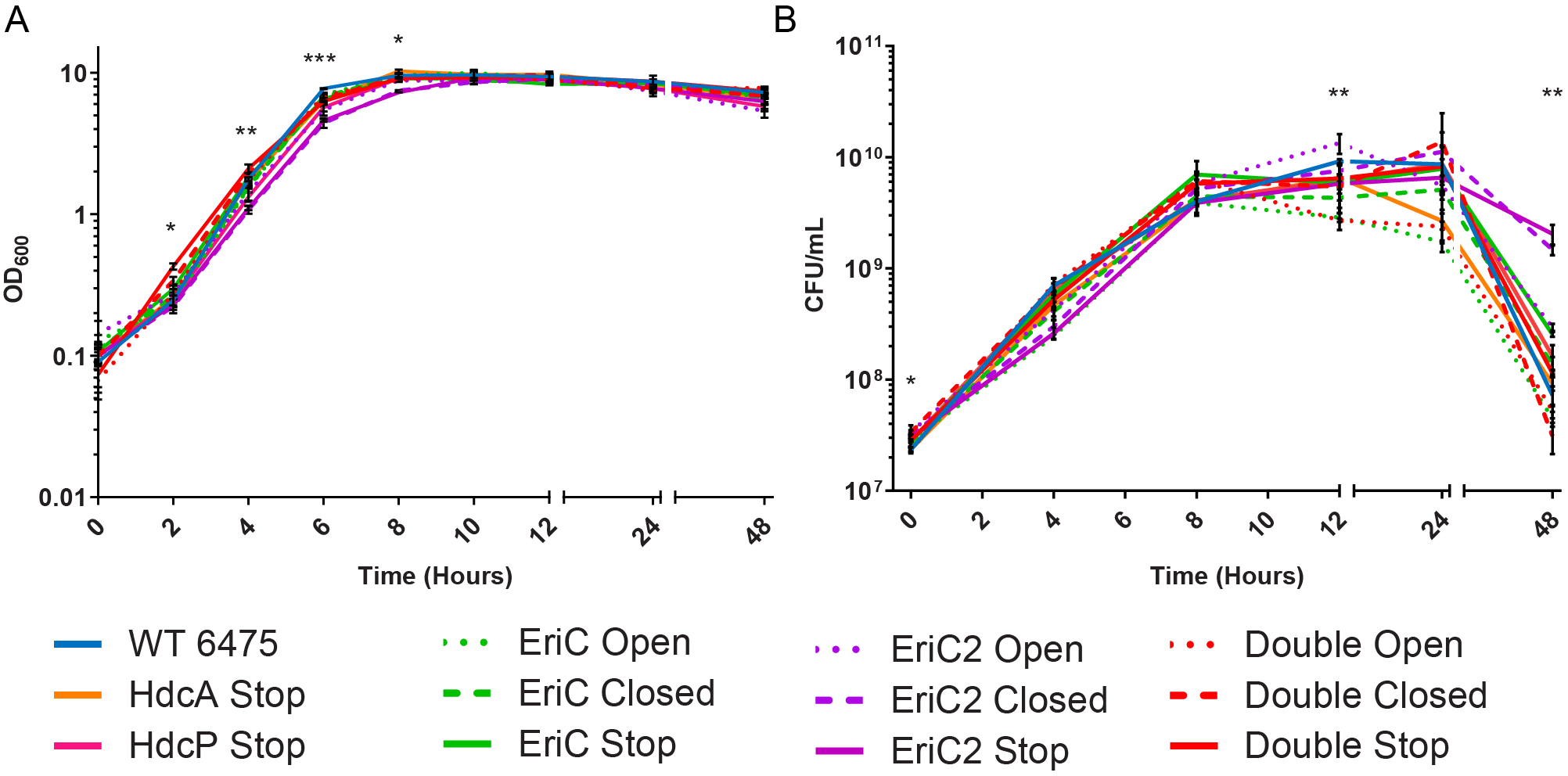

Supplement: Supplementary file 1 — Additional file 1: Figure S1. Growth parameters of L reuteri wild type 6475 and mutant strains in MRS medium over 48 h. Optical density (A) and viability (B) of bacterial cultures were measured at regular intervals during lag, exponential, and stationary phases. N = 3 per strain. Error bars represent ± SEM. Stars represent maximum P-values from a two-way repeated measures ANOVA with Dunnett’s multiple comparison tests performed within each timepoint relative to WT. [file 12934_2019_1264_MOESM1_ESM.tif]

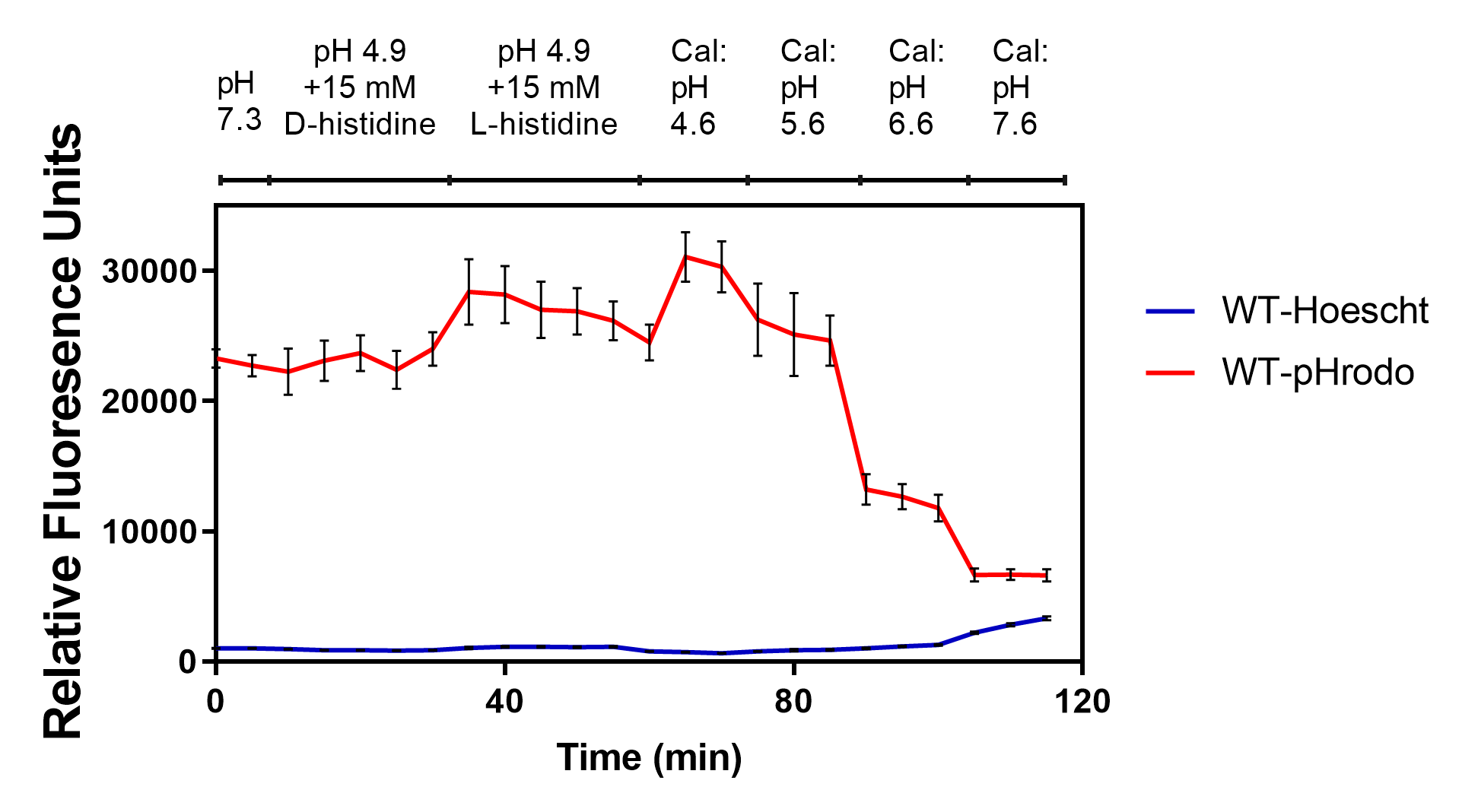

Supplement: Supplementary file 2 — Additional file 2: Figure S2. Comparison of pHrodo and Hoechst signals during intracellular pH assay procedure. Red trace indicates pHrodo fluorescence and blue trace indicates Hoechst fluorescence during a pilot intracellular pH assay. N = 6. Error bars represent ± SEM. The stability of the Hoechst signal throughout the assay suggest signal is not being lost due to cell lysis throughout the testing period. [file 12934_2019_1264_MOESM2_ESM.tif]
