# Supplementary material for: ClC transporter activity modulates histidine catabolism in Lactobacillus reuteri by altering intracellular pH and membrane potential
Source: Microb Cell Fact. 2019 Dec 12;18:212. doi: 10.1186/s12934-019-1264-0 (PMC6909576; doi:10.1186/s12934-019-1264-0)
Supplement: Supplementary file 3 — Additional file 3: Table S1. Description of bacterial strains used in this study. Table S2. A Buffer composition: variable pH histamine production assay (Fig. 1a). B Buffer composition: variable Cl− histamine production assay (Fig. 1b). Table S3. Description of genes and genomes used in in silico alignments in this study. Table S4. DNA oligonucleotides used in this study for mutagenesis. Table S5. Primers used in this study for screening and quantitative PCR. Table S6. Composition of non-commercial buffers used in the intracellular pH assay. Table S7. Composition of non-commercial buffers used in the membrane potential assay. [file 12934_2019_1264_MOESM3_ESM.docx]

**Table S1 –** Description of bacterial strains used in this study

| **Name** | **Strain Characteristics^1^** | **IMG Gene ID^2^** | **Ref.** |
| --- | --- | --- | --- |
| *Lactobacillus reuteri* ATCC PTA 6475 | Wild type | n/a | (42) |
| *L. reuteri* ATCC PTA 6475::pJP042 | Wild type strain carrying the pJP042 recombineering plasmid;  erythromycin-resistant | n/a | (66) |
| *L. reuteri* ATCC PTA 6475-HdcA Stop | Amino acid substitutions in the HdcA histidine decarboxylase at S83X and F84K convert serine to stop codon and phenylalanine to lysine; functional inactivation of HdcA | 2502291757 | Modeled from (17) |
| *L. reuteri* ATCC PTA 6475-HdcP Stop | Amino acid substitutions in the HdcP histidine/histamine antiporter at P25G and Q26X convert proline to glycine and glutamine to stop codon; functional inactivation of HdcP | 2502291758 | Modeled from (17) |
| *L. reuteri* ATCC PTA 6475-EriC Open | Amino acid substitution in the EriC H^+^/Cl^-^ antiporter at E144A converts glutamate to alanine at the internal gate; predicted to lose proton transport/allow gradient-dependent movement of chloride (28) | 2502290616 | This study |
| *L. reuteri* ATCC PTA 6475-EriC Closed | Amino acid substitution in the EriC H^+^/Cl^-^ antiporter at E199A converts glutamate to alanine at the external gate; predicted to abolish all ion movement through the transporter (34, 35) | 2502290616 | This study |
| *L. reuteri* ATCC PTA 6475-EriC Stop | Amino acid substitution in the EriC H^+^/Cl^-^ antiporter at Y13X, H14X, and K15X, converting tryptophan, histidine and lysine to stop codons; functional inactivation of EriC | 2502290616 | This study |
| *L. reuteri* ATCC PTA 6475-EriC2 Open | Amino acid substitution in the EriC2 H^+^/Cl^-^ antiporter at E139A converts glutamate to alanine at the internal gate; predicted to lose proton transport/allow gradient-dependent movement of chloride (28) | 2502290978 | This study |
| *L. reuteri* ATCC PTA 6475-EriC2 Closed | Amino acid substitution in the EriC2 H^+^/Cl^-^ antiporter at E193A converts glutamate to alanine at the external gate; predicted to abolish all ion movement through the transporter (34, 35) | 2502290978 | This study |
| *L. reuteri* ATCC PTA 6475-EriC2 Stop | Amino acid substitution in the EriC2 at E14X, R16X, and K17X, converts glutamate, arginine and lysine to stop codons; functional inactivation of EriC2 | 2502290978 | Modeled from (25) |

**Table S1 (continued) –** Description of bacterial strains used in this study

| *L. reuteri* ATCC PTA 6475-Dbl Open | Amino acid substitutions in EriC at E144A and in EriC2 at E139A converts glutamate to alanine at each of the internal gating positions of both H^+^/Cl^-^ antiporters | 2502290616  2502290978 | This study |
| --- | --- | --- | --- |
| *L. reuteri* ATCC PTA 6475-Dbl Closed | Amino acid substitution in EriC at E199A and in EriC2 at E193A converts glutamate to alanine at each of the the external gating positions of both H^+^/Cl^-^ antiporters | 2502290616  2502290978 | This study |
| *L. reuteri* ATCC PTA 6475-Dbl Stop | Amino acid substitution in EriC at Y13X, H14X, and K15X, and in EriC2 at E14X, R16X, and K17X converts tryptophan, histidine, lysine, glutamate, arginine and lysine to stop codons; functional inactivation of both H^+^/Cl^-^ transporters | 2502290616  2502290978 | This study |

^1^Standard single-letter amino acid notation is used to describe mutants; X indicates a stop codon.

^2^IMG gene accession information is provided for genes where mutations have been introduced.

**Table S2A** – Buffer Composition: Variable pH Histamine Production Assay (Figure 1A)

|  | **Reagent:** | | | |  | Osmolarity |
| --- | --- | --- | --- | --- | --- | --- |
|  | mMol Citric Acid | mMol K_2_HPO_4_ | mMol  L-Histidine HCl | mMol NaCl | mMol  KGluconate^1^ | (mOsm) |
| **Buffer:** |  |  |  |  |  |  |
| pH 7.5 | 6.35 | 187.30 | 15.00 | 80.00 | 0.00 | 758.25 |
| pH 6.5 | 27.25 | 145.50 | 15.00 | 80.00 | 52.25 | 758.25 |
| pH 5.5 | 42.00 | 116.00 | 15.00 | 80.00 | 89.13 | 758.25 |
| pH 4.5 | 53.25 | 93.50 | 15.00 | 80.00 | 117.25 | 758.25 |

^1^Potassium gluconate added as needed to achieve osmotic balance across buffer conditions

**Table S2B** – Buffer Composition: Variable Cl^-^ Histamine Production Assay (Figure 1B)

|  | **Reagent:** | | | |  | Osmolarity |
| --- | --- | --- | --- | --- | --- | --- |
|  | mMol  Citric Acid | mMol K_2_HPO_4_ | mMol  L-Histidine HCl | mMol NaCl | mMol  NaGluconate^1^ | (mOsm) |
| **Buffer**  **(pH 5.0):** |  |  |  |  |  |  |
| 15 mM Cl^-^ | 48.50 | 103.00 | 15.00 | 0.00 | 80.00 | 547.50 |
| 20 mM Cl^-^ | 48.50 | 103.00 | 15.00 | 5.00 | 75.00 | 547.50 |
| 35 mM Cl^-^ | 48.50 | 103.00 | 15.00 | 20.00 | 60.00 | 547.50 |
| 95 mM Cl^-^ | 48.50 | 103.00 | 15.00 | 80.00 | 0.00 | 547.50 |

^1^Sodium gluconate added as needed to achieve osmotic balance across buffer conditions

**Table S3** – Description of genes and genomes used in *in silico* alignments in this study

| **Name** | **IMG Genome ID^1^** | **IMG**  **Gene ID^2^** | **Genes Product Name** | **Gene Symbol** |
| --- | --- | --- | --- | --- |
| *Homo sapiens* | 639332100 | 639333861  639333864  639333833  639333834  639333835  639333836  639333837  639333855  639333856 | Chloride Channel Ka  Chloride Channel Kb  Chloride Channel 1  Chloride Channel 2  Chloride Channel 3  Chloride Channel 4  Chloride Channel 5  Chloride Channel 6  Chloride Channel 7 | CLCN-Ka  CLCN-Kb  CLCN1  CLCN2  CLCN3 CLCN4  CLCN5  CLCN6  CLCN7 |
| *Mus musculus* C57BL/6 | 639370800 | 639378300  639400420  639407721  639378294  639378295  639378296  639378297  639378298  639390296  639390297 | Chloride Channel Ka  Chloride Channel Kb  Chloride Channel K1  Chloride Channel 1  Chloride Channel 2  Chloride Channel 3  Chloride Channel 4  Chloride Channel 5  Chloride Channel 6  Chloride Channel 7 | Clcnka  Clcnkb  Clcnk1  Clcn1  Clcn2  Clcn3  Clcn4  Clcn5  Clcn6  Clcn7 |
| *Escherichia coli*  K-12 MG1655 | 2600254932 | 2600371447  2600372919  2600369228 | Chloride Channel EriC  Chloride Channel EriC  Chloride Channel EriC | ClcA  ClcB  YfeO |
| *Lactobacillus reuteri* ATCC PTA 6475 | 2502171170 | 2502290616  2502290978  2502290296  2502291937 | H_­­_^+^/Cl^-^ Antiporter ClcA  H_­­_^+^/Cl^-^ Antiporter ClcA  H_­­_^+^/Cl^-^ Antiporter ClcA  H_­­_^+^/Cl^-^ Antiporter ClcA | EriC  EriC2  *0247  *1843 |

^1^Sequences for *in silico* analysis were obtained from the Integrated Microbial Genomes (IMG) database (42).

^2^Relevant genes were found in the genomes of interest using the search term Cluster of Orthologous Groups 0038 (COG0038 - H^+^/Cl^-^ Antiporter) followed by manual curation of the results for those sequences possessing both the conserved internal and external gating domains.

*Prefix: IMG Locus Tag “HMPREF0536_”

**Table S4 -** DNA oligonucleotides used in this study for mutagenesis

| **Name** | **Target** | **Sequence^1^** | **Ref.** |
| --- | --- | --- | --- |
| oEriC_E144A | Convert E144A in EriC | GTTTGCTCACTATCTGTCCTGGACTTTTCTTAGGcaGaGccGGgCCAAGTATCCAAATTGGTGCTTGTATTGGAGCTTGC | This study |
| oEriC_E199A | Convert E199A in EriC | TCAGCGCTCCTTTAGCAGGAACAATGTTTTTgCTtGAgGccATGACGCATAATTTCAATTCCCGAATTTGGATTCCCGCT | This study |
| oEriC_Stop | Introduce early stop codons into EriC | TTGGAGGCAGTATTTGTGAAAAATGAAAATACCTTATAgtgatAACGGGCACGGGACATTTTAGGAAAACCATTCACCTC | Modeled from (25) |
| oEriC2_E139L_G140P | Convert E139L and G140P in EriC2 | CTCTACTCCTTGCCCAATTGTTGCCCCAAGTTGAATTGATGGTggaagACGTCCGAGATATAATCCGGATCCAATGGCGAAAATTCCACC | This study |
| oEriC2_L139A_P140G | Convert L139A and P140G in EriC2 | CTCTACTCCTTGCCCAATTGTTGCCCCAAGTTGAATTGATGGTccggcACGTCCGAGATATAATCCGGATCCAATGGCGAAAATTCCACC | This study |
| oEriC2_E193A | Convert E193A in EriC2 | TTTAATGCACCAATCGCTGCGACTATCTTTATTTTGGAgGccGTcTAcCATAATTTTTCACCGGTAATTTGGTTGGCAAC | This study |
| oEriC2_Stop | Introduce early stop codons into EriC2 | ATTGAACTGATTATAGCGATGAAATGCCTCTTaTCaAgTCTaCATTGTTTTTCTCCAATTTATTAAAAGTCTACATGCCA | This study |

^1^Lowercase letters indicate mutant sequence

**Table S5** – Primers used in this study for screening and quantitative PCR

| **Name** | **Target** | **PCR Type^1^** | **Sequence^2^** | **Tm°** | **Ref.** |
| --- | --- | --- | --- | --- | --- |
| EriC_F | EriC forward | MAMA-PCR | tgttgtaagtttttccgcaattt | 55°C | This study |
| EriC_R | EriC reverse | MAMA-PCR | gctgtaccaccaggtgaggt |  |  |
| EriC_M_E144A | EriC E144A mutant | MAMA-PCR | GACTTTTCTTAGGcaGaGccG |  |  |
| EriC_M_E199A | EriC E199A mutant | MAMA-PCR | AATGTTTTTgCTtGAgGccA |  |  |
| EriC_M_Stop | EriC stop mutant | MAMA-PCR | AAATACCTTATAgtgatAACGGGCAC |  |  |
| EriC2_F | EriC2 forward | MAMA-PCR | cgtcatggctttacaaacga | 49°C | This study |
| EriC2_R | EriC2 reverse | MAMA-PCR | gctttttgtccttcgctttg |  |  |
| EriC2_M_E139LG140P | EriC2 E139L G140P mutant | MAMA-PCR | TATCTCGGACGTcttccACC |  |  |
| EriC2_M_L139AP140G | EriC2 L139A P140G mutant | MAMA-PCR | ATTATATCTCGGACGTgccg |  |  |
| EriC2_M_E193A | EriC2 E193A mutant | MAMA-PCR | TCTTTATTTTGGAgGccGTcTAc |  |  |
| EriC2_M_Stop | EriC2 stop mutant | MAMA-PCR | tAGAcTtGAtAAGAGGCATTTCATCG |  |  |
| SG_rpoB_F | *rpoB* forward | qPCR | GCCTCTGCATGTATCCCATT | 60°C | (72) |
| SG_rpoB_R | *rpoB* reverse | qPCR | ATCATTGCAACCCCAGAGTC |  |  |
| SG_hdcA_F | *hdcA* forward | qPCR | CTTTGCTCATGCAATGATGG | 60°C | (72) |
| SG_hdcA_R | *hdcA* reverse | qPCR | ATCGAGCCACTTAGGCATTG |  |  |
| SG_hdcP_F | *hdcP* forward | qPCR | GCAATGGTTGGTTCAGGAGT | 60°C | (72) |
| SG_hdcP_F | *hdcP* reverse | qPCR | AATGAGCCAAATCCAACCTG |  |  |
| SG_eriC_F | *eriC* forward | qPCR | TCACCTGGTGGTACAGCAAA | 60°C | This study |
| SG_eriC_R | *eriC* reverse | qPCR | TGCCAATTCGTTGAAAGTGA |  |  |

**Table S5 (continued)** – Primers used in this study for screening and quantitative PCR

| SG_eriC2_F | *eriC2* forward | qPCR | GGACAGCACGGATTCCTAAA | 60°C | This study |
| --- | --- | --- | --- | --- | --- |
| SG_eriC2_R | *eriC2* reverse | qPCR | GCCCCACAAAAAGTGCTAAA |  |  |

^1^MAMA-PCR: Mismatch Amplification Mutation Assay- screening for mutant alleles; qPCR: SYBR green quantitative PCR

^2^Lowercase letters indicate a binding site for mutant sequence

**Table S6** – Composition of non-commercial buffers used in the intracellular pH assay.

|  | **Reagent:** | | | |  | Osmolarity |
| --- | --- | --- | --- | --- | --- | --- |
|  | mMol  Citric Acid | mMol K_2_HPO_4_ | mMol  Histidine HCl^1^ | mMol NaCl | mMol  KGluconate^2^ | (mOsm) |
| **Buffer:** |  |  |  |  |  |  |
| pH 4.9 | 48.50 | 103.00 | 15 (D-) | 80.00 | 105.375 | 758.25 |
| pH 4.9 | 48.50 | 103.00 | 15 (L-) | 80.00 | 105.375 | 758.25 |
| pH 4.6 | 53.25 | 93.50 | 15 (L-) | 80.00 | 117.25 | 758.25 |
| pH 5.6 | 42.00 | 116.00 | 15 (L-) | 80.00 | 89.125 | 758.25 |
| pH 6.6 | 27.25 | 145.50 | 15 (L-) | 80.00 | 52.25 | 758.25 |
| pH 7.6 | 6.35 | 187.30 | 15 (L-) | 80.00 | 0 | 758.25 |

^1^L-form or D-form of histidine used is indicated in parentheses

^2^Potassium gluconate added as needed for osmotic balance across buffer conditions

**Table S7** – Composition of non-commercial buffers used in the membrane potential assay

|  | **Reagent:** | | | |  | **Osmolarity** |
| --- | --- | --- | --- | --- | --- | --- |
|  | mMol  Na_2_HPO_4_ | mMol NaH_2_PO_4_ | mMol  NaCl | mMol Histidine^1^ | mMol  HCl | mOsm |
| **Potassium-free PBS:** |  |  |  |  |  |  |
| pH 7.1 | 7.00 | 3.00 | 140.00 | 0.00 | 0.00 | 300.00 |
|  |  |  |  |  |  |  |
| **Neutral Buffer:** |  |  |  |  |  |  |
| PBS, pH 7.3 | 7.00 | 3.00 | 140.00 | 15.00  (D-/L-) | 0.00 | 315.00 |
|  |  |  |  |  |  |  |
| **Acidic Buffer:** |  |  |  |  |  |  |
| PBS, pH 5.3 | 7.00 | 3.00 | 140.00 | 15.00  (D-/L-) | 15.15 | 330.15 |

^1^L-form or D-form of histidine used is indicated in parentheses
